# Degeneration of muscle spindles in a murine model of Pompe disease

**DOI:** 10.1038/s41598-023-33543-y

**Published:** 2023-04-21

**Authors:** Bridgette Watkins, Jürgen Schultheiß, Andi Rafuna, Stefan Hintze, Peter Meinke, Benedikt Schoser, Stephan Kröger

**Affiliations:** 1 Department of Physiological Genomics, Biomedical Center, Ludwig-Maximilians-University, Grosshaderner Strasse 9, 82152 Planegg-Martinsried, Germany; 2 Department of Neurology, Friedrich-Baur-Institute, LMU Klinikum, Ludwig-Maximilians-University, Munich, Germany

**Keywords:** Neuroscience, Physiology

## Abstract

Pompe disease is a debilitating medical condition caused by a functional deficiency of lysosomal acid alpha-glucosidase (GAA). In addition to muscle weakness, people living with Pompe disease experience motor coordination deficits including an instable gait and posture. We reasoned that an impaired muscle spindle function might contribute to these deficiencies and therefore analyzed proprioception as well as muscle spindle structure and function in 4- and 8-month-old *Gaa*^−/−^ mice. Gait analyses showed a reduced inter-limb and inter-paw coordination in *Gaa*^−/−^ mice. Electrophysiological analyses of single-unit muscle spindle proprioceptive afferents revealed an impaired sensitivity of the dynamic and static component of the stretch response. Finally, a progressive degeneration of the sensory neuron and of the intrafusal fibers was detectable in *Gaa*^−/−^ mice. We observed an increased abundance and size of lysosomes, a fragmentation of the inner and outer connective tissue capsule and a buildup of autophagic vacuoles in muscle spindles from 8-month-old *Gaa*^−/−^ mice, indicating lysosomal defects and an impaired autophagocytosis. These results demonstrate a structural and functional degeneration of muscle spindles and an altered motor coordination in *Gaa*^−/−^ mice. Similar changes could contribute to the impaired motor coordination in patients living with Pompe disease.

## Introduction

Pompe disease (OMIM #232300; glycogen storage disease II) is a rare, autosomal recessive, progressive, debilitating, and, in the case of the infantile onset form, lethal lysosomal storage disease^1–4^. The cause for Pompe disease are mutations in the *GAA* gene (MIM#606800), which codes for the lysosomal enzyme acid maltase (α-1,4-glucosidase; GAA; EC 3.2.1.20)^5^. The GAA enzyme catalyzes the degradation of glycogen and, accordingly, mutations, which affect the enzymatic activity of this enzyme, lead to an accumulation of glycogen in lysosomes^6^. This impairs lysosome function and leads to the degeneration of cells and entire organs^1^. The severity of the phenotype is proportional to the loss of enzyme activity^6^.

Although pathological lysosomal glycogen accumulation occurs in many tissues, skeletal and cardiac muscle are most prominently affected. Accordingly, patients living with Pompe disease show extensive fiber- and contractile apparatus degeneration, leading to progressive muscle weakness and atrophy, as well as varying degrees of respiratory complications due to dysfunction of the diaphragm and of the intercostal muscles. In addition to the muscular weakness, patients with Pompe disease experience impairments in executing motor abilities, including postural instability and an unstable gait, in particular when visual input is missing^7–9^. The cause of the impaired motor control is unknown. However, since there is almost no correlation between muscle weakness and postural parameters^9^, the motor control deficits are unlikely to be caused solely by degeneration and loss of skeletal muscle tissue.

Proprioceptive information informs the brain about the contractile status of our skeletal muscles and their force^10–12^. This information is required for the localization of our extremities in space and for the acquisition and execution of any coordinated movement, including walking and standing. Muscle spindles are the main proprioceptive sensors and are present in almost every muscle^12^. They consist of specialized muscle fibers (intrafusal fibers), which are grouped into nuclear bag and nuclear chain fibers, based on the number and arrangement of nuclei in their central (equatorial) region. Both types of intrafusal fibers are innervated in the equatorial region by group I and group II sensory afferents that generate action potentials, which are proportional to the length change as well as to the speed of stretching^13^. In addition, both ends of the intrafusal fibers (polar regions) are innervated by γ-motoneurons, which maintain the proprioceptive sensitivity of muscle spindles at all lengths and during all contraction phases of the skeletal muscle^14^.

In this study, we tested the hypothesis that an impaired muscle spindle function contributes to the motor control deficits, the instable gait and the frequent falls of Pompe disease patients. To this end, we structurally and functionally analyzed muscle spindles and locomotor behavior in 4- and 8-month-old *Gaa*^−/−^ mice^15^, which completely lack GAA enzymatic activity and have been used repeatedly as murine models for Pompe disease^16–20^. Our results show a reduced inter-limb and inter-paw coordination, a compromised response of muscle spindles to stretch and a severe degeneration of the sensory innervation, of the intrafusal fibers and of the muscle spindle outer capsule in 8-month-old *Gaa*^−/−^ mice. A considerably weaker phenotype was observed in 4-month-old *Gaa*^−/−^ mice. Collectively, these results demonstrate a progressively impaired muscle spindle structure and function as well as a reduced motor coordination in *Gaa*^−/−^ mice.

## Results

### ***Gaa***^−/−^ mice have motor coordination deficits

**Figure 1:**
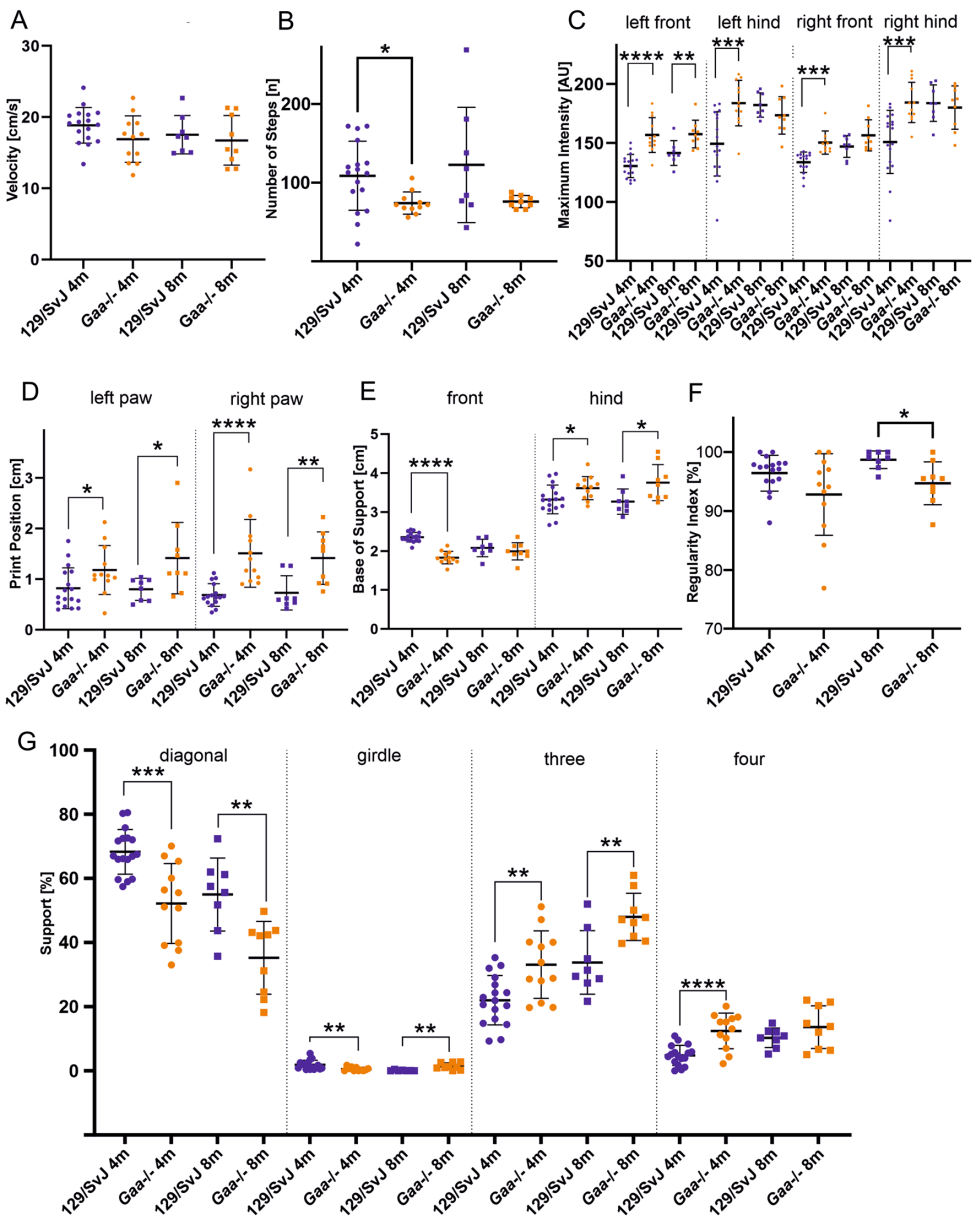
*Gaa*^−/−^ mice have an abnormal motor coordination. Automatic gait analysis revealed that many general locomotor parameters were similar in wildtype 129/SvJ mice (blue dots) and *Gaa*^−/−^ mice (orange dots), including velocity of movement (**A**) and number of steps (**B**; the slightly reduced number of steps in 4-month-old mice is most likely due to the weight difference). Other parameters are different between *Gaa*^−/−^ mice and age-matched control mice due to their different muscle force, including the maximum intensity of the footprints (**C**). On the other hand, both mouse lines behaved differently with respect to locomotion coordination parameters, including the print position (the distance between the position of the hind paw and the position of the previously placed front paw on the same side of the body and in the same step cycle; **D**), the base of support for the front- and hind limbs (**E**), as well as the regularity index (**F**). Moreover, the time the mice were supported by contacting the ground with the diagonal and girdle sides limbs as well as the time the animal was supported by three or four limbs was longer in *Gaa*^−/−^ mice compared to 129/SvJ control mice (**G**). For a complete list of gait parameters analyzed see Supplementary Table 1. The bars show the mean ± SD with N = 17 (4-month-old 129SvJ) and N = 12 (4-month-old *Gaa*^−/−^), N = 8 (8-month-old 129SvJ), N = 9 (8-month old *Gaa*^−/−^) mice. Statistical significance was calculated using the unpaired student’s t-test.

To investigate gait deficits in *Gaa*^−/−^ mice, we analyzed their motor behavior using the CatWalk XT system. This gait analysis system allows the automatic, quantitative and observer-independent investigation of a large number of dynamic and static movement parameters, which can be categorized into 4 major groups^21^: (a) run characteristics and kinetic parameters, (b) temporal parameters, (c) spatial parameters, and (d) interlimb coordination parameters (for a list of which parameter was categorized into which group see Supplementary Table 1). Many run characteristics and kinetic parameters assessing general gait and locomotor functions did not differ significantly between *Gaa*^−/−^ mice and age-matched control mice. These included velocity (measured as distance over time; Fig. 1A), body speed (calculated by dividing the distance that the animal’s body traveled from one initial contact of that paw to the next by the time to travel that distance; Supplementary Table 1) or stride length (distance between paw placement in two consecutive steps of the same paw; Supplementary Table 1). In contrast, from the ~ 200 parameters analyzed by the CatWalk XT system, 115 (4-month-old) and 83 (8-month-old) were significantly different between the *Gaa*^−/−^ mice and age-matched 129/SvJ control mice (Supplementary Table 1). Some of these differences are likely to be the result of the reduced muscle strength and different weight of the *Gaa*^−/−^ mice^16,20,21^. These include the number of steps per run in 4-month-old *Gaa*^−/−^ mice (Fig. 1B), stand time (duration of contact of a paw with the glass plate; Supplementary Table 1), maximum intensity of the paw pressure to the ground (Fig. 1C) or the print area (surface of the complete print; Supplementary Table 1). On the other hand, several differences in the interlimb coordination parameters^21^ between wildtype and *Gaa*^−/−^ mice are consistent with an impaired motor control. These parameters include the print position (distance between the position of the hind paw and the position of the previously placed front paw on the same side of the body (ipsilateral) and in the same step cycle; Fig. 1D), which was increased on both sides and at both ages in *Gaa*^−/−^ mice. In addition, the base of support (average width between two paws) particular of the hind limbs was increased (Fig. 1E). Likewise, the regularity index, defined as the exclusive use of normal step sequence patterns during uninterrupted locomotion (number of normal step sequence patterns relative to the total number of paw placements) was significantly lower in 8-month-old animals compared age-matched control mice (Fig. 1F), demonstrating that *Gaa*^−/−^ mice have more footprints outside of a recognized pattern.

The relative duration of the simultaneous contact with the glass plate of all combinations of paws is another parameter, which differed significantly between *Gaa*^−/−^ and control mice at both ages analyzed (Fig. 1G). None of the control or mutant mice had no paw on the glass plate at any time point during the run, and we observed no difference between *Gaa*^−/−^ and wildtype control mice in the percent of time where only a single paw had contact with the glass plate (Supplementary Table 1). In contrast, the time each animal was supported by simultaneous contact of the diagonal pair of paws (right front paw and left hind paw or left front paw and right hind paw) were significantly lower in *Gaa*^−/−^ mice compared to wildtype mice (Fig. 1G). The time of support for the girdle paws (right front paw and left front paw or right hind paw and left hind paw) was lower in 4-month-old and higher in 8-month-old *Gaa*^−/−^ mice (Fig. 1G). Moreover, the relative amount of time the animal simultaneously spent on three or four paws was higher in *Gaa*^−/−^ mice compared to age-matched control mice (Fig. 1G). The quantification of all approximately 200 parameters determined by the CatWalk system is summarized in Supplementary Table 1. Collectively, our results demonstrate an abnormal gait performance, locomotor function and particularly a compromised inter-limb- and inter-paw coordination in *Gaa*^−/−^ mice.

### Electrophysiological analysis of muscle spindles in *Gaa*^−/−^ mice

**Figure 2:**
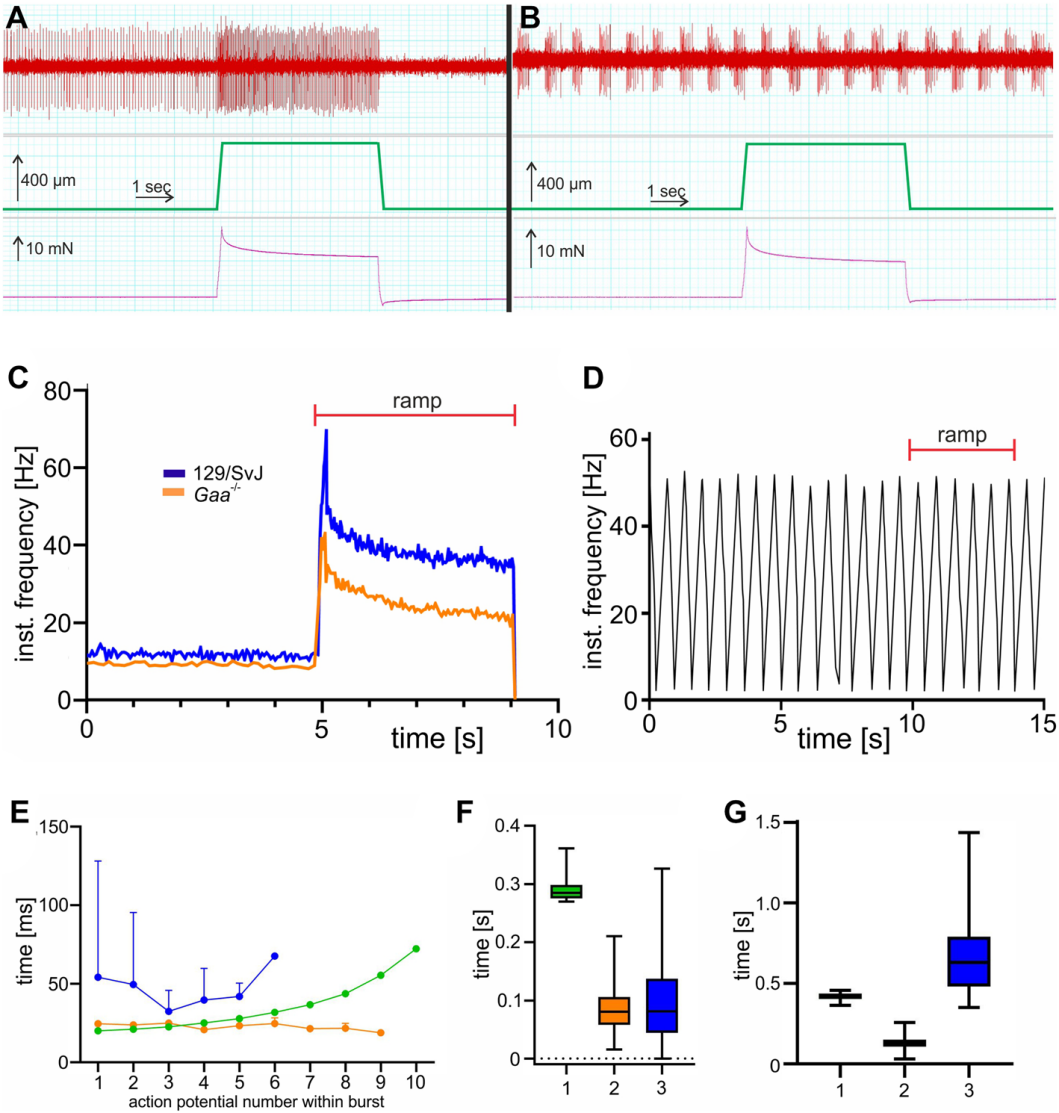
The response to stretch in muscle spindles from *Gaa*^−/−^ mice is impaired. A representative example for the response of muscle spindle afferents to a ramp-and-hold stretch from 129/SvJ wildtype mice is shown in panel (**A**). Muscle spindles from wildtype mice had a constant resting discharge of approximately 10 Hz and responded to stretch with an increase in their instantaneous frequency (**A**). In contrast, 70% of the muscle spindles from 8-month-old *Gaa*^−/−^ mice (orange line in panel (**C**)) responded to stretch, but had a lower frequency at all time points during a stretch compared to age-matched 129/SvJ wildtype mice. Approximately 30% of the 8-month-old *Gaa*^−/−^ mice fired bursts at rest and did not respond to stretch (**B**). In some spindles, the bursts were rather regular even during a ramp-and-hold stretch (**D**). In other muscle spindles from 8-month-old *Gaa*^−/−^ mice, which showed the bursting behavior at rest, the bursts varied with respect to the action potential frequency during the bursts (**E**), the duration of the bursts (**F**) and the interburst interval (duration of the silent period; (**G**)). Three representative spindles from three different 8-month-old *Gaa*^−/−^ mice are shown to illustrate the spectrum of the busts. The red bars in panels (**C,D**) indicate the duration of the ramp-and-hold stretch. The middle parts of panels (**A,B**) show the length change (5% of L_0_) and the lower parts show the passive tension generated by the muscle in response to the stretch. No difference between wildtype and *Gaa*^−/−^ mice was observed with respect to the passive tension generated in response to the length change.

**Figure 3:**
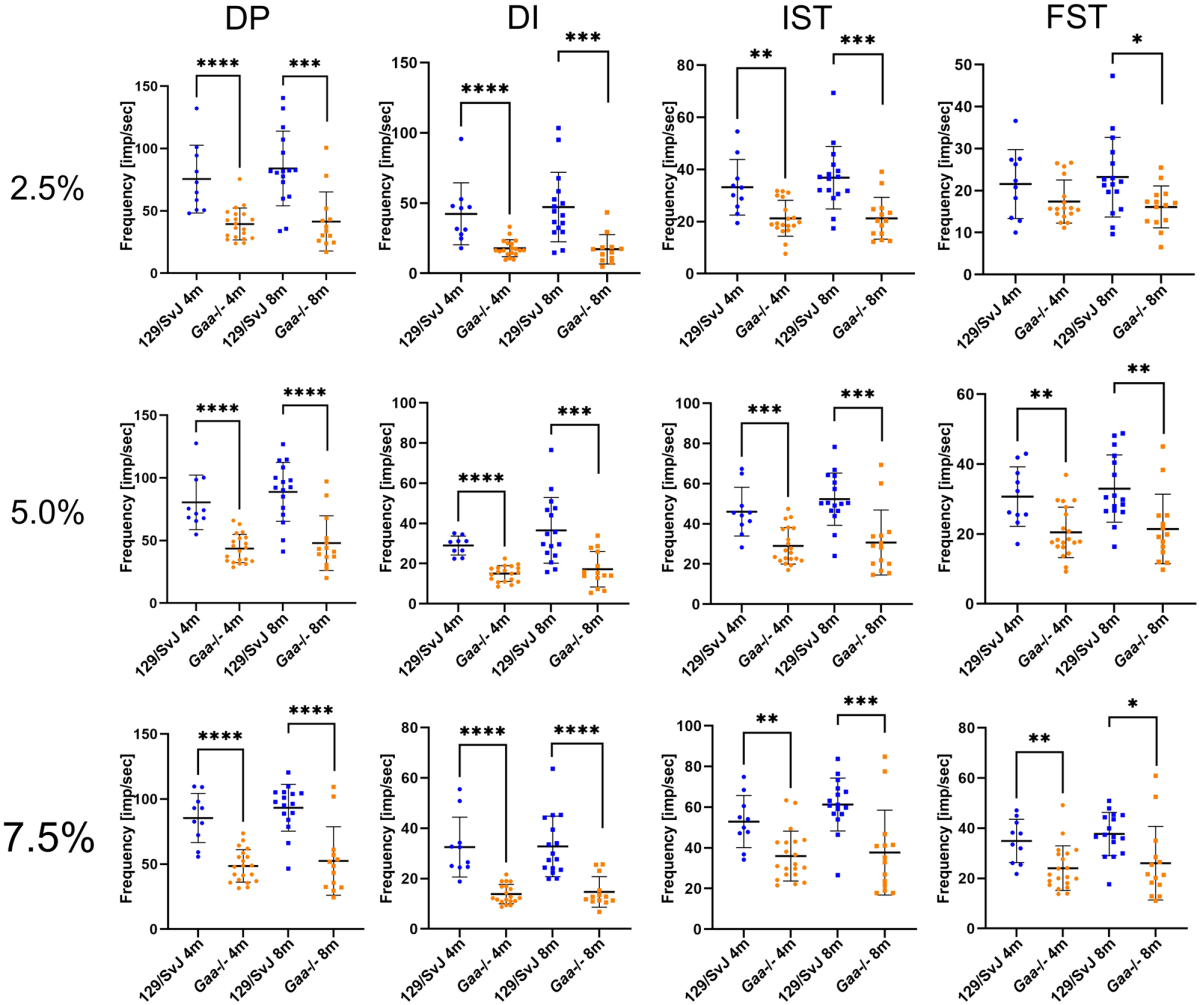
Stretch-responsive muscle spindles from *Gaa*^−/−^ mice have lower instantaneous frequencies compared to control mice at all time points during ramp-and-hold stretches. The frequency at four time points during a ramp-and-hold stretch (dynamic peak (DP), dynamic index (DI), initial static time (IST) and final static time (FST); for details of these time points see “Methods” section) were lower in *Gaa*^−/−^ mice (orange dots) compared to age-matched wildtype 129/SvJ control mice (blue dots). This was independent of the length change (2.5, 5.0 and 7.5% of L_0_) and of the age of the mice (4- and 8-month-old mice, respectively). Each dot represents the recording of a single muscle spindle. Bars show the mean ± SD with N = 4 (4-month-old 129SvJ), N = 8 (4-month-old *Gaa*^−/−^), N = 6 (8-month-old 129SvJ) and N = 16 (8-month-old *Gaa*^−/−^). Statistical significance was calculated using the unpaired student t-test.

To investigate changes in muscle spindle function in *Gaa*^−/−^ mice, we recorded single-unit proprioceptive afferent responses to different stretch protocols in an ex vivo preparation of the extensor digitorum longus (EDL) muscle from 4- and 8-month-old *Gaa*^−/−^ mice and compared them to age-matched 129/SvJ control mice. Responses to ramp protocols with length changes of 2.5, 5 and 7.5% L_0_ (with ramp speeds of 40% L_0_ per sec) were obtained and during each stretch response, four parameters were quantified: dynamic peak (DP), dynamic index (DI), initial static time (IST) and final static time (FST; for details on these parameters see “Methods” section). A representative recording from a control mouse-derived muscle spindle is shown in Fig. 2A. All 4- and 8-month-old wildtype as well as all 4-month-old and ~ 70% of the 8-month-old *Gaa*^−/−^ mice responded to stretch with an increase of the instantaneous action potential frequency (Fig. 2A,C). However, the frequencies of the response to the different stretches were significantly lower in *Gaa*^−/−^ mice of both ages (Fig. 2C). The quantification of the individual parameters at different hold lengths (2.5, 5 and 7.5% of L_0_) of *Gaa*^−/−^ mice compared to age-matched control mice is summarized in Fig. 3 (blue dots: 129/SvJ control mice; orange dots: *Gaa*^−/−^ mice). On average, the instantaneous frequencies at all four time points during all stretch protocols were lower in *Gaa*^−/−^ mice compared to 129/SvJ control mice, demonstrating a reduced static and dynamic sensitivity to stretch in *Gaa*^−/−^ mice.

In approximately 30% of the recordings from 8-month-old *Gaa*^−/−^ mouse muscle spindles at rest, we observed bursts of action potentials followed by short periods of silence without action potentials (Fig. 2B). In individual spindles, the bursts were rather regular (Fig. 2D and orange line in Fig. 2E). However, a more detailed analysis of the bursting behavior of three representative muscle spindles from three different mice demonstrated the variability of the bursts between different spindles with respect to the instantaneous frequency during the bursts (Fig. 2E), the duration of the bursts (Fig. 2F) and the length of the interburst interval (Fig. 2G), demonstrating a heterogeneity of the bursts in different muscle spindles. Most importantly, muscle spindle afferents with a bursting behavior did not respond to stretch with an increase of the instantaneous frequency but instead maintained the burst behavior throughout the stretch stimulus (Fig. 2B,D). These bursts were never observed in any of the control mice or in 4-month-old *Gaa*^−/−^ mice. In summary, these results demonstrate a severely compromised sensitivity to stretch of muscle spindles from 4- and 8-month-old *Gaa*^−/−^ mice and a complete insensitivity to stretch in ~ 30% of the recordings from 8-month-old *Gaa*^−/−^ mice.

### Structural degeneration of muscle spindles in *Gaa*^−/−^ muscle spindles

**Figure 4:**
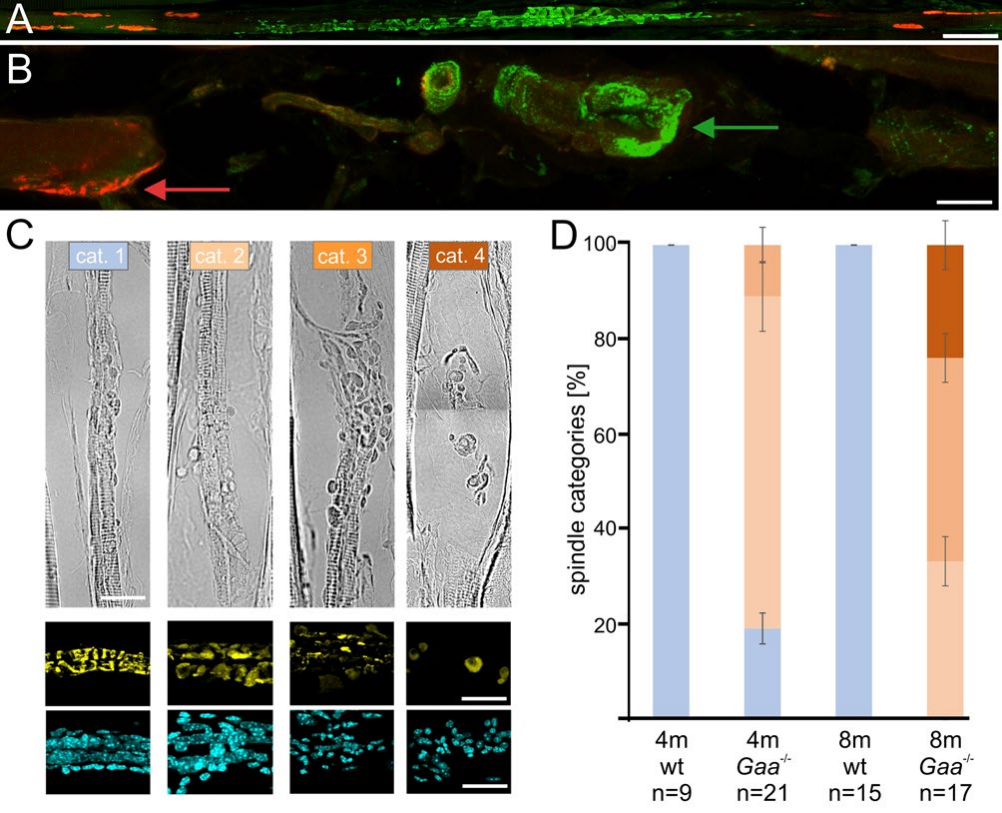
Quantification of the degenerative changes in muscle spindles from *Gaa*^−/−^ mice. Intrafusal fibers are innervated by sensory nerve terminals in the central (equatorial) region (stained with antibodies against vGluT1; green channel in (**A,B**)) and by γ-motoneurons in the polar regions, which form a cholinergic synapse (indicated by α-bungarotoxin labeling of the AChRs; red channel in (**A,B**)). Note the normal structure of the intrafusal fiber innervation in muscle spindles from 8-month-old 129/SvJ mice (**A**) and the severely degenerated innervation in the 8-month-old *Gaa*^−/−^ mice ((**B**); corresponding to a category 4 muscle spindle, see below). The sensory nerve terminal has retracted from the intrafusal fiber and has formed a large varicosity (green arrow). AChRs have disaggregated (red arrow) and the intrafusal fiber has lost its elongated shape and formed a spherical myoball-like structure. Panel (**C**) shows representative examples of the four different categories used to characterize the different levels of degeneration. For a more detailed description of the categories, see “Methods” section. The lower two rows of panel (**C**) show representative examples corresponding to the different categories of the morphology of the sensory ending (green channel) and the distribution of the nuclei (blue channel), respectively. Morphological analysis of 4- and 8-month-old muscle spindles from wildtype (wt) and *Gaa*^−/−^ mice revealed a progressive increase of the number of damaged muscle spindles demonstrating the progressive degenerative changes (panel (**D**)). Bars show the mean ± SD with N = 3; n represents the number of muscle spindles analyzed. Color-coding of the different categories is identical in panels (**C,D**). Scale bar: (**A**) 50 µm, (**B**) 20 µm, (**C**) 20 µm.

To investigate, if the muscle spindle functional deficits and the impaired movement coordination in *Gaa*^−/−^ mice were accompanied by structural changes, we compared the morphology of their muscle spindles with age-matched 129/SvJ mice using antibodies against several marker proteins. In adult muscle spindles, the vesicular glutamate transporter 1 (vGluT1) labels the sensory nerve terminal, which in mice revolves around the intrafusal muscle fibers in the form of an annulospiral ending^12^. This typical structure is visible in muscle spindles from 4- and 8-month-old wildtype animals (green channel in Fig. 4A and data not shown). Alpha-bungarotoxin labels nicotinic acetylcholine receptors (AChRs) predominantly at endplates of γ-motoneurons in the polar regions of the intrafusal fibers (red channel in Fig. 4A). The distribution of both, α-bungarotoxin and vGluT1, were severely altered in muscle spindles from 8-month-old *Gaa*^−/−^ mice (Fig. 4B). The sensory nerve terminal had retracted from the intrafusal fiber, had lost its annulospiral morphology and had formed large varicosities (green arrow in Fig. 4B). The intrafusal fibers had also degenerated and often formed a round, myoball-like structure with patchy AChR clusters on its surface (red arrow in Fig. 4B).

Since the extent of degeneration of muscle spindles was rather heterogeneous even within the same muscle, we quantified the structural changes by defining four categories of progressively severe structural damage of muscle spindles (see “Methods” section for more details; Fig. 4C). Quantification showed that 4- and 8-month-old 129/SvJ control mice had muscle spindles without any detectable damage (category 1; Fig. 4D). In contrast, in 4-month-old *Gaa*^−/−^ mice, only ~ 20% of the muscle spindles had a normal structure, whereas ~ 70% were mildly (category 2) and ~ 10% severely affected by degenerative processes (Fig. 4D). We observed no normal muscle spindle in 8-month-old *Gaa*^−/−^ mice and about 65% of all spindles were either severely damaged (category 3) or had completely deteriorated (corresponding to category 4; Fig. 4D). Consistently, we observed a reduction of the total number of muscle spindles in the soleus muscle from 8.3 ± 0.96 in wildtype to 4.7 ± 0.58 (mean ± SD with N = 3) in 8-month-old *Gaa*^−/−^ mice, respectively. These results demonstrate a progressive structural degeneration and a reduction of muscle spindle number in *Gaa*^−/−^ mice.

**Figure 5:**
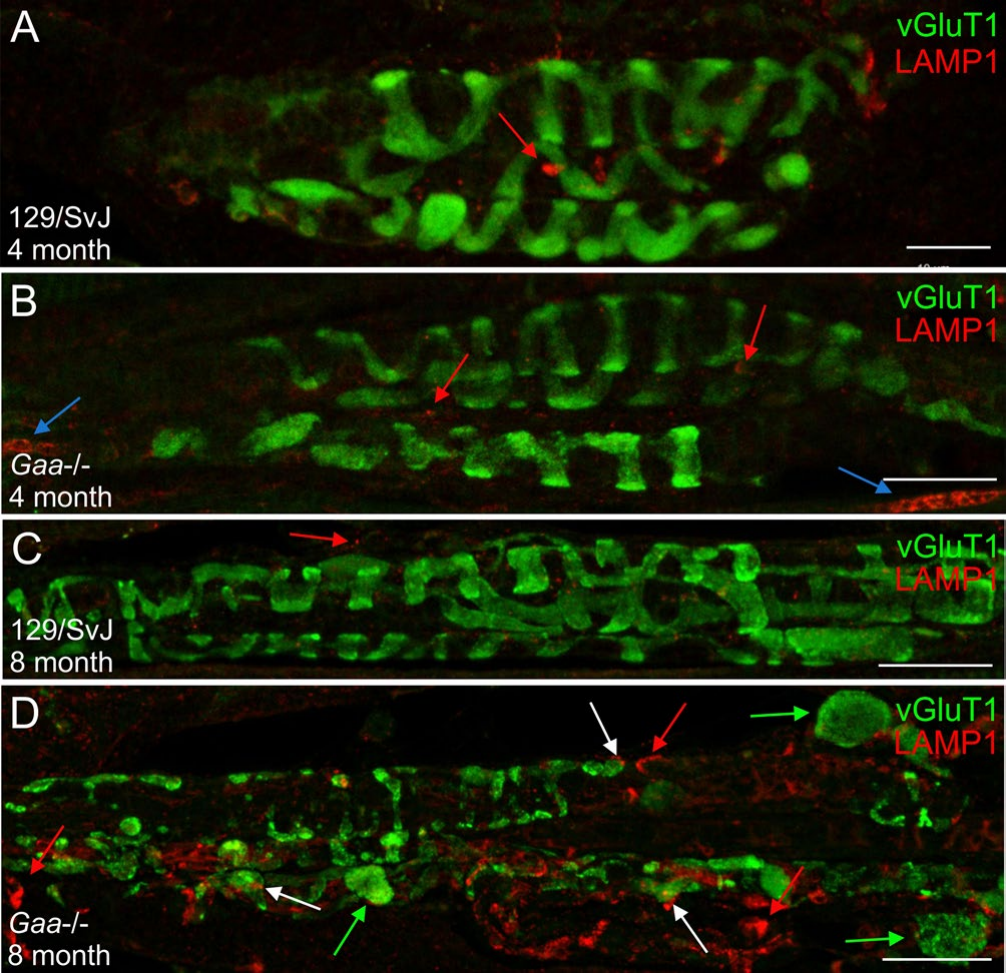
Accumulation and aggregation of lysosomes in muscle spindles from *Gaa*^−/−^ mice. The structure and distribution of lysosomes was investigated using anti-LAMP1 antibodies in 4-month-old (**A,B**) and 8-month-old (**C,D**) 129/SvJ control mice (**A,C**) and *Gaa*^−/−^ mice (**B,D**). The structure of the sensory nerve terminal (stained by antibodies against vGluT1; green channel) was indistinguishable in 4- and 8-month-old muscle spindles from wildtype mice and in 4-month-old *Gaa*^−/−^ mice. In contrast, in 8-month-old *Gaa*^−/−^ mice (category 2), the sensory nerve terminal had formed several varicosities within the spindle matrix (green arrows in panel (**D**)), had retraced from the intrafusal fibers and had lost the typical annulospiral morphology. Lysosomes were barely detectable in sections from 129/SvJ mice at 4- and 8 months of age (red arrows in panels (**A,C**)). In contrast, lysosomes were significantly enlarged and had formed aggregates in 4-month-old *Gaa*^−/−^ mice (red and blue arrows in panel (**B**), respectively). By 8 months (panel (**D**)), lysosomes were abundant, highly aggregated (red arrows) and often associated with the varicosities formed by the sensory nerve terminals of *Gaa*^−/−^ mice (white arrows). Scale bars: 20 µm.

In extrafusal muscle fibers from Pompe disease patients and from *Gaa*^−/−^ mice, lysosomes are enlarged and their number is increased^2,22^. To investigate if intrafusal fibers show a similar change in lysosome size and number, we stained muscle spindles with antibodies against the lysosomal membrane protein LAMP1. We observed very few lysosomes in 4- and 8-month-old wildtype muscle spindles (red arrows in Fig. 5A,C). In contrast, small LAMP1-positive puncta and a few enlarged lysosomes were detectable in 4-month-old *Gaa*^−/−^ muscle spindles (red and blue arrows in Fig. 5B, respectively). In 8-month-old *Gaa*^−/−^ mice, anti-LAMP1 antibodies strongly stained aggregated lysosomes (red arrows in Fig. 5D), often associated with the varicosities formed by the sensory nerve terminal (white arrows in Fig. 5D). To quantify these changes, we determined the total number of pixels above threshold per area as well as the size of the lysosomal aggregates. This quantification revealed a non-significant increase of 38% (p = 0.2304) in the total number of pixels above threshold in 4-month-old *Gaa*^−/−^ mice compared to age-matched 129/SvJ mice. In contrast, the total number of pixels above threshold was significantly increased (p = 0.000002) in 8-month-old *Gaa*^−/−^ mice by 320%. Likewise, the size of the pixel aggregates was non-significantly increased by 22.8% (p = 0.4920) in 4-month-old *Gaa*^−/−^ mice. In 8-month-old animals, the size of the pixel aggregates was significantly higher (p = 0.0078) in *Gaa*^−/−^ mice by 86.58% compared to age-matched 129/SvJ control mice. Consistent with previously published results^23^, an increase in the LAMP1 staining was also observed in extrafusal fibers from 4- and 8-month-old *Gaa*^−/−^ mice, suggesting a parallel increase in lysosomes in extra- and intrafusal fibers. These results demonstrate an increase in the number and in the size of LAMP1-positive lysosomes in muscle spindles from 8-month-old *Gaa*^−/−^ mice.

**Figure 6:**
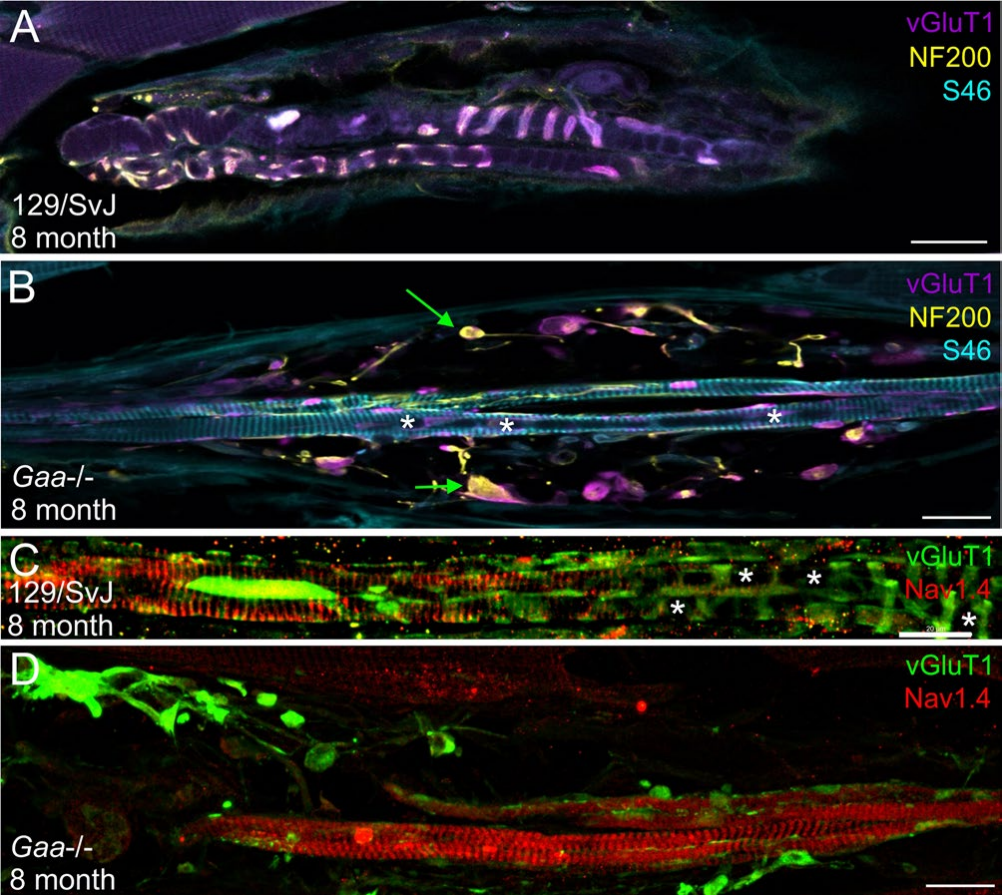
The varicosities formed by the sensory nerve terminal contain cytoskeletal elements and proteins of the polar region redistribute into the central region of intrafusal fiber’s in 8-month-old *Gaa*^−/−^ mice. Muscle spindles from 8-month-old wildtype (**A,C**) and *Gaa*^−/−^ (**B,D**) mice were stained with antibodies against vGluT1 (purple channel in panels A and B and green channel in panels (**C,D**)), neurofilament 200 (NF 200; yellow channel in (**A,B**)), the myosin heavy chain 6 (S46 antibody; blue channel in (**A,B**)) and the voltage-gated sodium channel Na_v_1.4 (red channel in (**C,D**)). In muscle spindles from wildtype 129/SvJ mice, neurofilament 200 immunoreactivity codistributed with the sensory nerve terminal (labeled by the anti-vGluT1 antibodies). The typical annulospiral morphology of the nerve terminal is detectable. In contrast, in muscle spindles from age-matched *Gaa*^−/−^ mice (panels (**B,D**) show category 3 muscle spindles), the contact between the sensory nerve terminal and the intrafusal fibers was lost and the sensory nerve terminals had retracted to form numerous varicosities. The varicosities labeled by vGluT1 contained neurofilament 200 immunoreactivity (green arrows in panel (**B**)), demonstrating the accumulation of cytoskeletal elements in these structures. Note the absence of labeling with the S46 antibody in the central region of 8-month-old wildtype intrafusal fibers (**A**) and its presence in the central region of intrafusal fibers from age-matched *Gaa*^−/−^ mice (**B**). Likewise, the voltage-gated sodium channel Na_v_1.4 was concentrated underneath the subsarcolemmal plasma membrane in the central region of intrafusal fibers of wildtype animals (**C**) whereas it was present throughout the intrafusal fiber in 8-month-old *Gaa*^−/−^ mice (**D**). Asterisks in panels (**B,C**) indicate the central region of intrafusal fibers. Scale bars: 20 µm.

Degenerating axons in the central and peripheral nervous system (for example after spinal cord injury or peripheral nerve damage) form varicosities at their distal ends, which are filled with cellular debris such as disorganized cytoskeletal elements^24^. To investigate if the varicosities observed in muscle spindles from 8-month-old *Gaa*^−/−^ mice contain cytoskeletal elements (and might therefore indicate degeneration of the sensory nerve terminal), we stained muscle spindles with antibodies against neurofilament 200. In 8-month-old wildtype animals, neurofilament immunoreactivity was observed in the sensory nerve ending, overlapping with the vGluT1 staining of the annulospiral endings of the sensory terminal (Fig. 6A). In contrast, in age-matched *Gaa*^−/−^ mice, neurofilament was disorganized and concentrated in the large vGluT1-positive varicosities (green arrows in Fig. 6B), suggesting that the sensory nerve terminals had degenerated and withdrawn from the intrafusal fiber and had aggregated neurofilament protein inside the varicosities.

Staining with antibodies against the myosin heavy chain 6 (S46 antibody; light blue channel in Fig. 6A,B) revealed few contractile filaments in the central region of intrafusal fibers in 8-month-old wildtype animals, in agreement with the almost complete absence of sarcomeres in this region (Fig. 6A^25^). In contrast, S46 staining was readily observed throughout the central region of intrafusal fibers in category 3-like muscle spindles from age-matched *Gaa*^−/−^ mice (Figs. 6B, 8D), demonstrating a redistribution of this myosin heavy chain in the central region of intrafusal fibers.

To confirm the redistribution of proteins in the central region of intrafusal fibers, we compared the distribution of the voltage-gated sodium channel Na_v_1.4 in 8-month-old wildtype and *Gaa*^−/−^ mice. The immunoreactivity for this channel is coextensive with the phalloidin-labeled actin filaments in intrafusal muscle fibers from wildtype mice^25^, and therefore an indicator of the contractile apparatus. Similar to the myosin heavy chain, we observed a concentration of Na_v_1.4 immunoreactivity specifically in the subsarcolemmal compartment of the central region of intrafusal fibers in wildtype muscle spindles (Fig. 6C^25^), but a presence of this channel throughout the intrafusal fiber, including the equatorial region, in 8-month-old *Gaa*^−/−^ mice (Fig. 6D). This demonstrates that in the central region of intrafusal fibers from 8-month-old *Gaa*^−/−^ mice, Na_v_1.4 and the myosin heavy chain 6 similarly redistribute.

**Figure 7:**
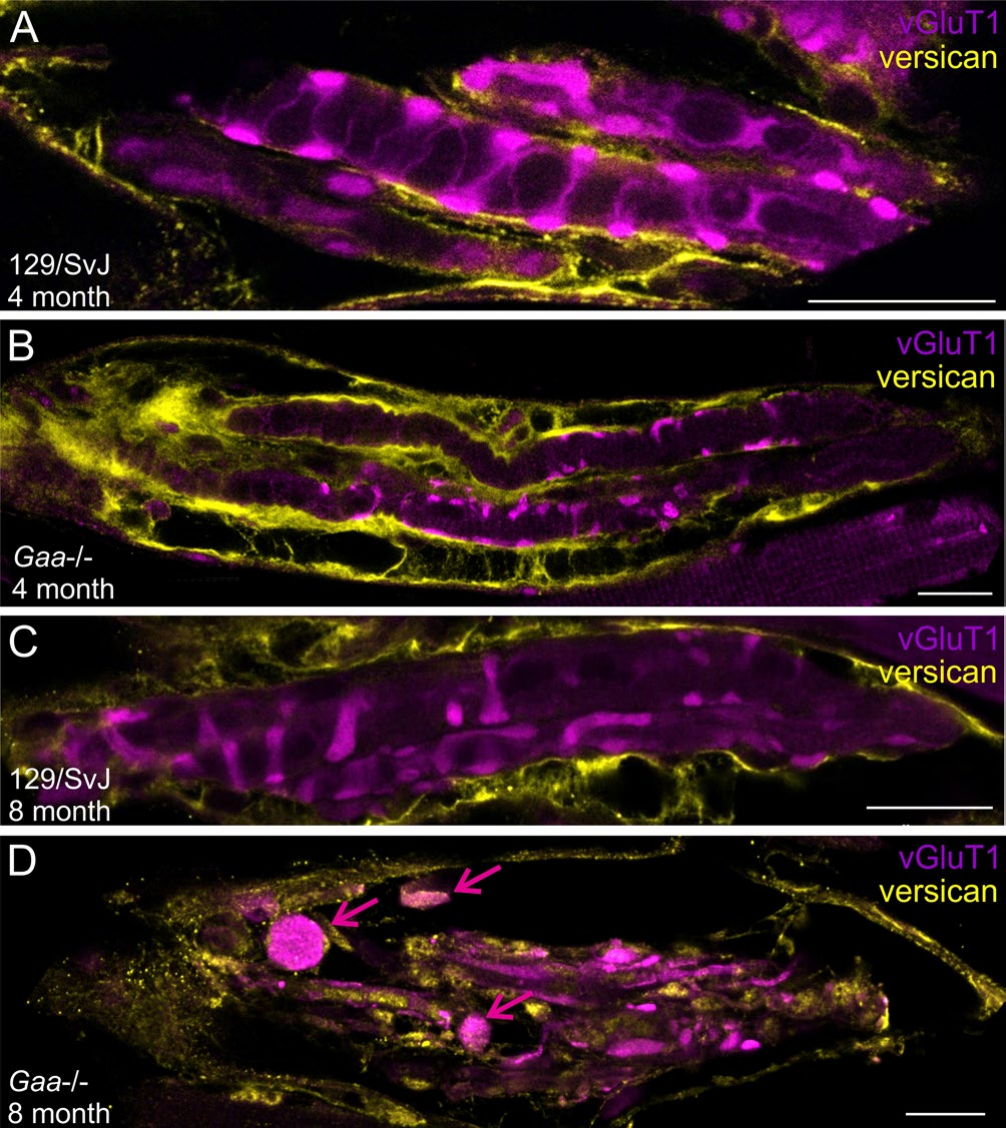
The muscle spindle connective tissue capsule deteriorates in 8-month-old *Gaa*^−/−^ mice. Muscle spindles from 4- (**A,B**) and 8-month-old (**C,D**) wildtype (**A,C**) and *Gaa*^−/−^ (**B,D**) mice were stained with antibodies against vGluT1 (purple channel) and the extracellular matrix protein versican (yellow channel). The antibodies labeled the inner and outer capsule of muscle spindles in wildtype and 4-month-old *Gaa*^−/−^ mice. In 8-month-old *Gaa*^−/−^ mice, the labeling was distributed throughout the muscle spindle and the inner- and outer capsule appeared fragmented. Note the vGluT1-positive varicosities (purple arrows in panel (**D**)) and the punctate versican immunoreactivity in the 8-month-old *Gaa*^−/−^ mice. Scale bar: 20 µm.

To investigate the extracellular matrix and the integrity of the connective tissue capsule of muscle spindles from wildtype and mutant animals, we compared the distribution of the matrix protein versican in *Gaa*^−/−^ mice and in age-matched 129/SvJ control mice. In skeletal muscle, anti-versican antibodies selectively label the muscle spindle extracellular matrix^26^. Versican immunoreactivity was restricted to the inner and outer capsule in muscle spindles from wildtype mice (Fig. 7A,C) and in 4-month-old *Gaa*^−/−^ mice (Fig. 7B). In contrast, it appeared throughout the spindle in a punctate pattern in 8-month-old *Gaa*^−/−^ mice (Fig. 7D). Moreover, 8-month-old muscle spindles showed signs of degeneration of the inner and outer capsule indicated by the fragmentation of the capsule-associated anti-versican staining (Fig. 7D). These results demonstrate an altered distribution of the versican immunoreactivity in 8-month-old *Gaa*^−/−^ mice, consistent with a degeneration of the extracellular matrix and the connective tissue capsule.

**Figure 8:**
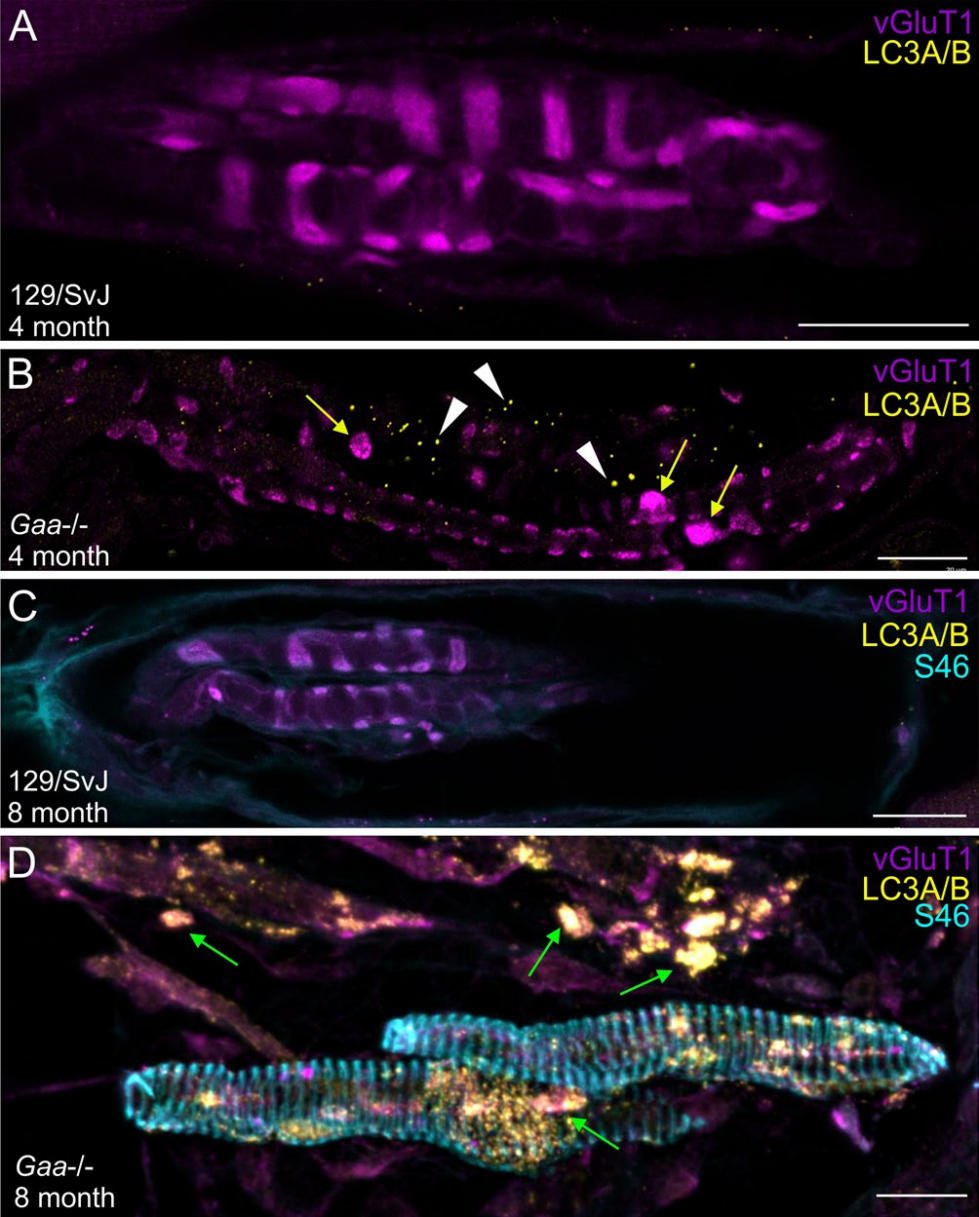
Autophagosomes accumulate in muscle spindles from *Gaa*^−/−^ mice. Muscle spindles from 4- (**A,B**) and 8-month-old (**C,D**) wildtype (**A,C**) and *Gaa*^−/−^ (**B,D**) mice were stained with antibodies against vGluT1 (purple channel) and the autophagosome marker LC3A/B (yellow channel). Muscle spindles from 8-month-old wildtype and *Gaa*^−/−^ mice were additionally stained with antibodies against myosin heavy chain 6 (S46 antibody; blue channel in panels (**C,D**)). While autophagosomes were mostly absent in muscle spindles from 4- and 8-month-old wildtype mice, they appeared in 4-month-old *Gaa*^−/−^ mice (white arrowheads in panel (**B**); category 2 muscle spindle). In 8-month-old *Gaa*^−/−^ mice autophagosomes were abundant (arrows in panel (**D**); category 3 muscle spindle) and the LC3A/B immunoreactivity was associated with intrafusal fibers (arrows in panel (**D**)) and with the varicosities formed by the sensory nerve terminal (green arrows in panel (**D**)). Note the formation of small varicosities by the sensory nerve terminal in muscle spindles from 4-month-old *Gaa*^−/−^ mice (yellow arrows in panel (**B**)). S46 immunoreactivity was absent in the central region of intrafusal fibers of 8-month-old 129/SvJ mice (**C**), but present in age-matched *Gaa*^−/−^ mice (**D**), confirming the reorganization of the intrafusal contractile filaments. Scale bars: 20 µm.

Lysosomal dysfunction in Pompe disease leads to incomplete autophagic flux and accumulation of autophagic debris, particularly in extrafusal fibers^23,27,28^. To investigate if autophagy is also initiated in muscle spindles from *Gaa*^−/−^ mice, we used antibodies against the LC3A/B protein, a well-established marker for autophagosomes^29^. We observed no obvious autophagic vacuoles in muscle spindles from 4- and 8-month-old wildtype mice (Fig. 8A,C). In contrast, autophagosomes were detectable in muscle spindles from 4-month-old *Gaa*^−/−^ mice as punctate immunoreactivity with anti-LC3A/B antibodies (white arrowheads in Fig. 8B). In 8-month-old muscle spindles from *Gaa*^−/−^ mice, autophagosomes were enlarged, aggregated and often associated with the intrafusal fibers and with the large varicosities formed by the sensory nerve terminal (green arrows in Fig. 8D). Quantification of mice of both genotypes and age revealed a significant (p = 0.0003) increase of 202% of the total number of pixels above threshold in 4-month-old *Gaa*^−/−^ mice. Likewise, the total number of LC3AB-positive pixels above background in 8-month-old *Gaa*^−/−^ animals was significantly (p = 0.0001) increased by 160%. Moreover, the size of the pixel aggregates was significantly increased (p = 0.0004) by 227% in 4-month-old *Gaa*^−/−^ mice. Likewise, in 8-month-old *Gaa*^−/−^ mice, the size of the pixel aggregates was significantly (p = 0.0010) increased by 111.90%. These results demonstrate a buildup of autophagosomes in muscle spindles from *Gaa*^−/−^ mice, and suggest that in these mice an abnormal autophagy might contribute to the severe degenerative processes of the muscle spindles.

Collectively, our immunohistochemical analyses demonstrate a severe and progressive degeneration of the sensory nerve terminal, the intrafusal fiber and the connective tissue capsule in muscle spindles from *Gaa*^−/−^ mice.

## Discussion

The *Gaa*^−/−^ mouse line recapitulates many of the hallmarks of patients living with Pompe disease^20,30,31^ and is therefore well suited to study the effect of a lack of GAA enzymatic activity on motor performance and muscle spindle structure and function. Using this mouse line, we provide three independent lines of evidence for a progressively impaired muscle spindle structure and function: (1) the dynamic and static response of single unit proprioceptive afferents to stretch is severely compromised, (2) muscle spindles from 8-month-old *Gaa*^−/−^ mice show severe signs of degeneration of intrafusal fibers, sensory nerve terminals and of the spindle capsule and, finally, (3) the locomotor behavior of these mice revealed an abnormal limb- and paw coordination as well as gait problems. Our results therefore suggest that degenerating muscle spindles could contribute to the gait instability, the frequent falls and the movement coordination deficits of patients living with Pompe disease.

The loss of contact of the sensory nerve terminals to the intrafusal fibers as well as the formation of varicosities and the accumulation of cytoskeletal elements in the varicosities of *Gaa*^−/−^ mice is typical for an axonal “dying back mechanism”^24^. Similar events have been described in muscle spindles from the gracile axonal dystrophy mouse line^32^. This structural disorganization together with the presence of autophagosomes suggest a preponderance of degenerative events occurring in muscle spindles but the underlying mechanism for the degeneration is unclear. One possibility is that the degeneration of the intrafusal fibers causes the degeneration of the sensory nerve terminal. It has been previously shown that sensory nerve terminals require the secretion of neurotrophin-3, which activates the tropomyosin receptor kinase C (TrkC) receptor on proprioceptive sensory neurons and by this secures the survival of the sensory neuron^33–35^. Thus, the degeneration of intrafusal fibers could secondarily cause the degeneration and retraction of the proprioceptive sensory axon. On the other hand, glycogen deposits have also been observed in the peripheral nervous system and in dorsal root ganglia neurons of patients with Pompe disease^18,20,36–38^. Accordingly, these patients develop a polyneuropathy^38^ and a loss of peripheral nerves, leading among others to peripheral areflexia^9,39^**.** Since the continuous secretion of neuregulin-1 by the sensory neuron is required for intrafusal fiber survival^40^, the degeneration of the intrafusal fibers might be secondary to the degeneration of the sensory terminal. Finally, it is also possible that the degeneration of the sensory neuron and of the intrafusal fibers are independent processes occurring in parallel. In any case, the result would be a degeneration of the entire muscle spindle. If this degeneration is causal to the proprioceptive deficits, including uncoordinated movements and an abnormal gait, however, remains to be shown by a more detailed study.

Degenerating muscle spindles are not well characterized on the molecular level. We therefore used the *Gaa*^−/−^ mice to investigate the subcellular distribution of a number of molecules during the degeneration process. Among others, we observed that several proteins usually almost exclusively present in both polar regions of intrafusal fibers redistributed into the central (equatorial) region of intrafusal fibers in 8-month-old *Gaa*^−/−^ mice. For example, the voltage-gated sodium channel Na_v_1.4 was present in the central region of wildtype muscle spindles only as a thin layer underneath the plasma membrane^25^. In contrast, it appeared throughout the intrafusal fiber in degenerating muscle spindles from 8-month old *Gaa*^−/−^ mice. Similarly, the nuclear bag fiber-specific myosin heavy chain 6 (stained by the S46 antibody) labeled only the subsarcolemmal sarcomeres in the central region of wildtype intrafusal fibers^25^ but was present throughout the central part of intrafusal fibers in 8-month-old *Gaa*^−/−^ mice. This suggests that intrafusal fibers change the subcellular distribution of their cytoskeleton and of associated molecules in degenerating muscle spindles. The altered distribution of the contractile apparatus was observed in the predominantly fast twitch EDL muscle and in the predominantly slow twitch soleus muscle. Therefore, the degeneration and the changes in the distribution of the contractile apparatus did not apparently depend on the muscle’s fiber composition. It will be interesting to investigate if the innervation by the sensory nerve regulates the sarcomere distribution in intrafusal fibers.

Electrophysiological analyses of the muscle spindle response to ramp-and-hold stretches revealed severe defects in *Gaa*^−/−^ mice, i.e. the dynamic as well as the static components of the stretch response were significantly reduced. These deficits were present already in 4-month-old mice and we therefore consider them the first functional signs of the muscle spindle degeneration. In contrast, ~ 30% of the recordings from 8-month-old *Gaa*^−/−^ mice did not show a constant resting discharge but instead fired in bursts and did not respond to stretch. Since we only observed bursting behavior in 8-month-old *Gaa*^−/−^ mice, it is likely that it represents a later stage of the degeneration process. One possibility to explain these bursts is that the generation of high frequency action potentials requires a continuous availability of glucose, which in the absence of GAA enzymatic activity might not be abound. Increasing the glucose content in the ACSF bathing solution, however, did not affect the bursting behavior. Moreover, the bursts could be recorded for several hours with only little changes in their duration and in their interburst interval, suggesting that they might reflect long-term axonal changes and are not the result of an acute glucose deficiency or damage. Since a similar spontaneous bursting behavior has been observed previously in human muscle spindle afferents during postischemic or mechanically-induced paresthesiae^41,42^, these bursts might reflect the degeneration of the axon and of its myelin sheath. The consequences of the reduced static and dynamic sensitivity and of the failed response to stretch would be a severely compromised proprioception, consistent with the abnormal motor coordination observed in 8-month-old *Gaa*^−/−^ mice.

The muscle spindles with a bursting behavior did not respond to stretch. Since stretch sensitivity together with the spindle pause after the stretch are essential criteria for the identification of proprioceptive afferents, we do not have proof that the neurons with the bursting behavior are proprioceptive sensory afferents. However, since they appeared only in 8-month-old *Gaa*^−/−^ mice and since their action potential frequencies were in the same range as that of proprioceptive afferents, and since similar bursting behavior has been described previously in sensory afferents from damaged muscle spindles, we consider it likely that they represent proprioceptive afferents, which had degenerated to an extend not allowing them to respond to stretch.

While the majority of muscle spindles from 8-month-old *Gaa*^−/−^ mice displayed severe signs of structural degeneration with, for example, an absence of normal annulospiral structure of the sensory terminal, approximately 70% were still able to respond to stretch, albeit at reduced instantaneous frequencies. The reason for this discrepancy is unclear, but it should be noted that in our electrophysiological experiments, we are strongly biased towards muscle spindles with a stretch response and completely non-functional spindles would not have been detected. It is therefore possible that in our electrophysiological analyses, the few remaining functional muscle spindles are overrepresented. It should also be considered, that proprioceptive sensory neurons from damaged muscle spindles might still be able to rudimentarily respond to stretch even without direct contact to intrafusal fibers. A more detailed study directly relating structural degeneration of muscle spindles with altered responses to stretch is required to causally link both processes.

Previous studies have shown several motor symptoms in *Gaa*^−/−^ mice including a reduced activity in an open field environment, symptoms of skeletal muscle deterioration, including an abnormal waddling gait, muscle weakness, poor performance in the rotarod test and remarkably different footprints^16^. Most of these deficits were detected in aged (> 12 month old) animals and directly or indirectly reflect the progressive skeletal muscle weakness of these animals. Our study, analyzing 4- and 8-month-old mice (presumably with less severe symptoms and representing an early symptomatic stage), represents the first systematic analysis of the limb- and paw coordination in this model organism for Pompe disease. We observed significant deficits particularly in inter-limb and inter-paw coordination. It is tempting to speculate that these deficits are caused by the degeneration of muscle spindle structure and function. However, interpreting the gait analysis requires caution, since some differences in the motor behavior are likely to be explained by the progressive degeneration of skeletal muscle tissue in *Gaa*^−/−^ mice. Moreover, we compared *Gaa*^−/−^ mice, which have a mixed C57BL/6 and 129/J background, with inbred 129/SvJ mice and, therefore, some of the differences could be due to the strain differences. However, transection of the dorsal column in rat (selectively eliminating the proprioceptive input to the CNS without affecting weight), resulted in similar motor coordination and gait changes as observed in *Gaa*^−/−^ mice^43^, demonstrating that these parameters can reflect proprioceptive deficits. Additionally, the print area was significantly increased across all paws in the *Gaa*^−/−^ mice, even though their body weight is less compared to the 129/SvJ control mice. A more detailed study is required to demonstrate that the functional and structural degeneration of the muscle spindles causes the gait impairment and that the altered gait is caused by an altered proprioception.

Late-onset Pompe disease patients exhibit a number of motor coordination deficits, including for example decreased velocity and cadence, increased stance phase, increased time of double limb support, shorter step and stride length as well as a wider base of support^7^. The print position results, increased stance time and increase in hind base of support in *Gaa*^−/−^ mice are similar to the motor symptoms in patients. Differences in the mutant mice between the front- and hindlimbs (including for example the wider base of support) are likely explained by the fact that the hindlimbs are outside of the visual field preventing a visual compensation of the motor coordination deficits^44^. The fundamental differences between biped humans and quadruped mice, however, make a direct translation of *Gaa*^−/−^ mouse gait abnormalities to humans difficult^7–9^.

We were not able to directly analyze the accumulation of glycogen in muscle spindles from *Gaa*^−/−^ mice due to the incompatibility of the histochemical staining for glycogen and the immunohistochemical staining required to identify muscle spindles. However, in an autopsy study of a single Pompe disease patient, glycogen accumulation in intrafusal fibers from several different muscles has been reported^45^. Consistent with a glycogen accumulation-based degeneration of muscle spindles, Pompe disease patients present with reduced gait velocity, cadence, time in single stand and other abnormalities during posturographic analysis^9^. These symptoms together with the loss in muscle strength lead to an increased risk of falls, hospitalization and as a result in muscular atrophy due to immobilization^46,47^. Our study suggests that muscle spindle deficits might contribute to the posturographic symptoms and the frequent falls of Pompe patients. Proprioceptive training^48,49^, including whole body vibration training with an oscillating platform^50,51^, should therefore be incorporated into the Pompe disease therapy to improve proprioception and reduce the risk of injury and hospitalization.

## Methods

### Animals and muscle preparation

Experiments were performed on muscles from *Gaa*^−/−^ mice (B6;129-*Gaa*^*tm1Rabn*^/J; The Jackson Laboratories, strain 004154), originally generated by Raben et al.^15^. In these mice, exon 6 of the *GAA* gene was targeted with a termination codon and a neomycin cassette leading to a complete absence of the GAA enzyme in these mice. Mice of both sexes were tested at an age of 16 to 18 weeks (4-month-old) or between 34 and 36 weeks (8-month-old), respectively. Age- and sex-matched 129/SvJ mice (129X1/SvJ; The Jackson Laboratories; strain 000691) were used as controls in all experiments. At 16 to 18 weeks of age, the *Gaa*^−/−^ mice have a reduced mobility and strength particularly in vertical motion and an accumulation of lysosomes in extrafusal fibers^15,16,52^. At 8 months of age, the *Gaa*^−/−^ mice develop obvious muscle wasting, a weak waddling gait and a decline in motor performance and coordination^15,16,20^. In agreement with the literature, we observed no difference between male and female mice in our experiments and no change in the body weight between *Gaa*^−/−^ and wildtype control mice at 8 month of age^22,53^. A total of 52 animals (8 wildtype and 9 *Gaa*^−/−^ mice were used for immunocytochemistry and 10 wildtype and 25 *Gaa*^−/−^ mice for electrophysiology) were used in this study. All animal procedures used in this study were performed according to the guidelines from Directive 2010/63/EU of the European Parliament on the protection of animals used for scientific purposes. The study is reported in accordance with ARRIVE (Animal Research: Reporting of In Vivo Experiments) guidelines (https://arriveguidelines.org). Experimental protocols were designed to minimize the number of experimental animals. All experiments were approved by the local authorities of the State of Bavaria, Germany (Az.: ROB-55.2-2532.Vet 02-17-82).

### Locomotor behavior

The gait of twelve 4-month-old and nine 8-month old *Gaa*^−/−^ mice was compared with 17 and 8, respectively, age-matched 129/SvJ control mice using the CatWalk XT system (Noldus Information Technology, Wageningen, Netherlands^21,54^). This system allows the observer-independent quantitative analysis of several movement parameters, including speed of locomotion, symmetry of leg use as well as paw and digit position^54–56^. Animals were brought to the testing room 7 days before the commencement of the experiments. Before each experiment, mice were acclimatized to the walkway and the dark testing room for 5 min per day for 1 week. The experiments were conducted according to the manufacturer’s suggestions and always at the same time of the day (between 10am and 1 pm). Each traverse of the walkway is termed a “run”. All runs for a given animal are termed a “trial”. Three consecutive compliant runs per trial were averaged and three trials for every mouse were performed. Each animal was tested individually, and food was placed in a goal-orientated box. Incomplete or non-compliant runs (below or above the set run duration of 0.5 to 5 s) were not scored. The same detection settings were used for all mice (Camera Gain: 9.64, Green Intensity Threshold: 0.11, Red Ceiling Light: 17.8, and Green Walkway Light: 19.00). After all test animals were analyzed, the raw data were exported as an Excel file for further analysis by the CatWalk XT software (version 10.6, Noldus Information Technology).

For the analysis of the ~ 200 parameters, we categorized them according to Ref.^21^ into 4 major groups: (1) run characteristics and kinetic parameters, (2) temporal parameters, (3) spatial parameters, and (4) interlimb coordination parameters. The first three categories are more sensitive to muscle strength, locomotion speed and body weight^21,57^, whereas the last category is considered proprioception-related and therefore the parameters of this group were analyzed in more detail. These included base of support, print position and regularity index.

### Electrophysiology

Afferent sensory neuron responses to stretch were assayed using an isolated muscle–nerve preparation previously described^58–61^. Ten muscle spindles from four 4-month-old and 16 muscle spindles from six 8-month-old 129/SvJ mice were investigated and compared to 21 muscle spindles from eight 4-month-old and 22 muscle spindles from fourteen 8-month-old *Gaa*^−/−^ mice, respectively. In brief, mice were sacrificed by cervical dislocation to avoid an interference of the anesthetic with the sensory afferent recordings. The extensor digitorum longus (EDL) muscle together with the deep peroneal branch of the sciatic nerve were then dissected and placed in a 25 ml in vitro tissue bath (809B-IV, Aurora Scientific, Dublin) containing oxygenated artificial cerebrospinal fluid (ACSF^58^). The tendons at one end were sutured to a fixed post and at the other end to a lever arm, connected to a dual force and length controller (300C-LR; Aurora Scientific, Dublin, Ireland) allowing the simultaneous recording of muscle tension and muscle length. Sensory activity was sampled using a suction electrode (tip diameter 50–70 μm) connected to an extracellular amplifier (Model 1800, A&M Systems, Elkhart, USA). A signal was classified as being from a putative muscle spindle afferent if it displayed a characteristic instantaneous frequency response to stretch as well as a pause during twitch contraction^58–60^. Baseline muscle length (L_0_) was defined as the minimal length at which maximal twitch contractile force was generated. For every muscle spindle afferent recording, triplicates of 10 s resting discharge followed by ramp-and-hold stretches (L_0_ plus 2.5, 5 or 7.5% of L_0_; ramp speed 40% L_0_ s^−1^; ramp phase duration: 0.1 s; hold phase: 3.8 s; stretch duration: 4 s with 45 s intervals between each stretch^61^) were recorded and averaged. From these recordings the dynamic peak (DP; highest firing rate during ramp), the dynamic index (DI; firing rate of dynamic peak − initial static time), the initial static time (IST; firing rate 0.45–0.55 s into stretch) and the final static time (FST; firing rate 3.25–3.75 s into stretch) were determined^25,60,62^ and compared to the same values from age-matched 129/SvJ control mice.

At the end of each recording, general muscle health was ensured by determining the maximal contractile force during a direct tetanic stimulation (500 ms train at 120 Hz frequency and 0.5 ms pulse length, supramaximal voltage; Grass SD9 stimulator; Natus, Pleasanton, USA^58,60^). This value was normalize for differences in muscle size and mass by determining the diameter of the EDL muscle at L_0_. With this information, the specific force (force/cross-sectional area) was determined in wildtype and *Gaa*^−/−^ mice and compared to the previously reported peak force of the EDL of 23,466 N/cm^2^^63,64^. We observed no statistically significant difference in the specific force between wildtype and *Gaa*^−/−^ mice at both ages analyzed.

For data analysis, action potentials from individual sensory neurons were identified by spike shape and the discriminator view using the Spike Histogram feature of LabChart (v8.1.5; ADInstruments, Sydney, Australia). Action potentials from additional potential muscle spindles that appeared during the stretch (detectable by a different frequencies and amplitudes) were not scored. No attempt was made to discriminate group Ia from group II afferents (see Wilkinson et al.^58^ for a detailed discussion).

### Statistical analysis

Differences between the means of the action potential frequencies and the gait parameters were compared statistically using student’s unpaired t-test. All statistical analyses were performed using GraphPad Prism (version 9.3.1). The level of significance (p-value) for all statistical tests was set at * < 0.05, ** < 0.01, *** < 0.001 and **** < 0.0001.

### Immunocytochemistry

Immunofluorescence labelling was performed as described previously^25,60,61,65^. To obtain muscle tissue for immunohistochemistry, mice were deeply anaesthetized via an I.P. injection of ketamine (100 mg kg^−1^; Pfizer, Berlin, Germany) and xylazine (10 mg kg^−1^; Bayer AG, Leverkusen, Germany). After transcardial perfusion with PBS followed by 4% paraformaldehyde, the soleus, the gastrocnemius, the tibialis anterior and EDL muscles were dissected. Fixed muscles were embedded in Tissue-Tek O.C.T. Compound (Sakura Finetek Europe, AJ Alphen an den Rijn, Netherlands), rapidly frozen and cryo-sectioned along the longitudinal axis at 20–30 μm thickness.

Dried frozen sections were rehydrated for 10 min in PBS. Sections were then blocked in PBS containing 0.2% Triton X-100 (Sigma-Aldrich Chemie GmbH, Taufkirchen, Germany) and 1% bovine serum albumin (Carl Roth GmbH, Karlsruhe, Germany; blocking solution) for 60 min at room temperature and incubated with the primary antibody in blocking solution at 4 °C overnight.

Sensory nerve terminals were identified using antibodies from guinea pig against the vesicular glutamate transporter 1 (vGluT1; AB5905, Millipore, Darmstadt, Germany; 1:1000)^60,65^. The S46 monoclonal antibody (diluted 1:50) against the slow tonic myosin heavy chain 6 developed by F. Stockdale^66^ was obtained from the Developmental Studies Hybridoma Bank, created by the NICHD of the NIH and maintained at The University of Iowa (Department of Biology, Iowa City, IA 52242)^25,67–69^. Neurofilament was detected using antibodies against NF200 (N4142, Sigma-Aldrich, Darmstadt, Germany; 1:500). To investigate autophagosomes, an antibody against LC3A/B was used (PAI-16931; Thermo Fisher Scientific-Invitrogen, Waltham, USA; 1:500)^29^. Antibodies against the lysosomal membrane glycoprotein LAMP1 (L1418, Sigma-Aldrich, Darmstadt, Germany; 1:500), which plays an important role in lysosome biogenesis and autophagy, were used to investigate lysosomal buildup. Versican was detected using a rabbit anti-versican antibody (Ab19345; Abcam, Cambridge, UK; 1:500^26^). The distribution of the voltage-gated sodium channel was analyzed using a polyclonal rabbit antibody (SCN4A; #ASC-020; Alomone labs, Jerusalem, Israel; 1:500^25^).

Primary antibodies were detected using the appropriate Alexa488-, Alexa594- and Alexa647-conjugated goat anti-rabbit (A11034; Thermo Fisher Scientific-Invitrogen, Waltham, USA; 1:1000), goat anti-guinea pig (A11076; Thermo Fischer Scientific-Invitrogen; 1:1000) or goat anti-mouse (A32723; Thermo Fischer Scientific-Invitrogen 1:1000) secondary antibody. To detect false positive results due to unspecific binding of antibodies, negative controls (without primary antibodies or with normal goat serum as primary antibodies) were stained in parallel. No specific labelling was observed under these conditions.

After immunofluorescence labelling, the sections were embedded in Aqua Polymount (18606; Polymount, Hirschberg, Germany) and analyzed using a Zeiss LSM 710 laser scanning confocal microscope (Carl Zeiss AG, Oberkochen, Germany) as previously described^25,59–61^. We observed no obvious morphological difference between muscle spindles of the EDL, the soleus, the tibialis anterior or the gastrocnemius muscle in mice of the same age and genotype. Therefore, the structural data of muscle spindles from all muscles of the same age and genotype were pooled and compared to pooled data from age-matched mice with a different genotype. Quantification of the number of muscle spindles per soleus muscle was performed as described previously^61^. The soleus muscle was chosen since it is a small muscle and since the number of muscle spindles has been analyzed previously^61,70,71^. Three soleus muscles each from a different wildtype or 8-month-old *Gaa*^−/−^ mouse, respectively, were reconstructed.

Digital processing of entire images, including adjustment of brightness and contrast, was performed using Photoshop CS6 (Adobe Inc., San Jose, USA). Compound images were assembled using CorelDraw (vs 19.1.0.419; Corel Corporation, Ottawa, Canada).

To quantify the progression of the degenerative processes in muscle spindles from different ages, four different categories were defined, which can be distinguished in cryostat sections using brightfield microscopy (see Fig. 4A for representative examples of the categories):

- *Category 1 (no degeneration)*: normal structure of the muscle spindle, complete circumferential elements of the annulospiral endings, intrafusal fibers in close proximity to each other and normal distribution of nuclei typical for nuclear bag and nuclear chain fibers, few sarcomeric structures in the central region of intrafusal fibers, no varicosities formed by sensory terminal.
- *Category 2 (mild degeneration)* Sensory terminals have formed few varicosities, circumferential elements present but often not continuous, sections with more than 8 nuclei in a row in typical nuclear bag and nuclear chain fiber arrangement detectable, intrafusal fibers partially detached from each other.
- *Category 3 (severe degeneration)* several large varicosities formed by sensory terminal, severe degradation of circumferential parts of the annulospiral endings, circumferential elements are mostly disrupted, sarcomeric structures abundant in central region of intrafusal fiber, nuclei evenly distributed within capsule and no nuclear arrangement typical for nuclear bag and chain fibers detectable, intrafusal fibers are separated by a large space.
- *Category 4 (completely deteriorated)* Sensory nerve terminal completely absent or only detectable as a large varicosity with no circumferential elements, no intrafusal fiber present, no sarcomeric structures detectable, capsule filled with cellular debris, outer capsule swollen, nuclei pycnotic.

To quantify the immunofluorescence signal detected in muscle spindles with antibodies against LAMP1 and LC3AB, muscle spindles from the EDL, the soleus and the tibialis anterior were analyzed. Since we did not observe obvious differences between muscle spindles form different muscles with respect to the categories described above, results from all muscles of the same age and genotype were pooled. Sections were stained as detailed above and images were acquired as z-stacks using the same scanning speed and averaging. Using the ZEN software (vs. 3.5 blue edition, Carl Zeiss Microscopy GmbH, Göttingen, Germany) z-stacks were orthogonally projected using maximum intensity of the frontal plane (XY) and the background was subtracted from all images. The channels were split and pixels above threshold in the appropriate channel were counted unbiased using ImageJ (version 1.53q^72^). Thresholding was kept consistent across all images. The “Analyze Particles” function was used to calculate the total number of pixels above threshold in a defined area and the sum of the pixels above threshold in an identified particle (“size of particle”). Results were expressed as percent of control. The following number of animals (N) and number of spindles (n) were analysed: Four-month-old 129/SvJ animals: N = 4, n = 8; 8-month-old control animals: N = 3, n = 5; 4-month-old *Gaa*^−/−^ animals: N = 3, n = 6; 8-month-old *Gaa*^−/−^ animals: N = 3, n = 6. Statistical significance was calculated using the unpaired student’s T-test in Excel (Microsoft Corporation, Redmond, USA).

### Supplementary Information

Supplementary Table 1.

## Data Availability

The datasets generated during and/or analyzed during the current study are available from the corresponding author on reasonable request.

## References

[1] van der Ploeg AT, Reuser AJ (2008). Pompe's disease. Lancet.

[2] Lim JA, Li L, Raben N (2014). Pompe disease: From pathophysiology to therapy and back again. Front. Aging Neurosci..

[3] Kohler L, Puertollano R, Raben N (2018). Pompe disease: From basic science to therapy. Neurotherapeutics.

[4] Peruzzo P, Pavan E, Dardis A (2019). Molecular genetics of Pompe disease: A comprehensive overview. Ann. Transl. Med..

[5] Martiniuk F, Bodkin M, Tzall S, Hirschhorn R (1991). Isolation and partial characterization of the structural gene for human acid alpha glucosidase. DNA Cell Biol..

[6] De Filippi P (2014). Genotype-phenotype correlation in Pompe disease, a step forward. Orphanet J. Rare Dis..

[7] McIntosh PT, Case LE, Chan JM, Austin SL, Kishnani P (2015). Characterization of gait in late onset Pompe disease. Mol. Genet. Metab..

[8] Valle MS (2016). Quantitative analysis of upright standing in adults with late-onset Pompe disease. Sci. Rep..

[9] Schneider I (2020). Characterization of gait and postural regulation in late-onset Pompe disease. Appl. Sci..

[10] Proske U, Gandevia SC (2012). The proprioceptive senses: Their roles in signaling body shape, body position and movement, and muscle force. Physiol. Rev..

[11] Kröger S (2018). Proprioception 2.0: Novel functions for muscle spindles. Curr. Opin. Neurol..

[12] Kröger S, Watkins B (2021). Muscle spindle function in healthy and diseased muscle. Skelet. Muscle.

[13] De-Doncker L, Picquet F, Petit J, Falempin M (2003). Characterization of spindle afferents in rat soleus muscle using ramp-and-hold and sinusoidal stretches. J. Neurophysiol..

[14] Banks RW (1994). The motor innervation of mammalian muscle-spindles. Prog. Neurobiol..

[15] Raben N (1998). Targeted disruption of the acid alpha-glucosidase gene in mice causes an illness with critical features of both infantile and adult human glycogen storage disease type II. J. Biol. Chem..

[16] Raben N, Nagaraju K, Lee E, Plotz P (2000). Modulation of disease severity in mice with targeted disruption of the acid alpha-glucosidase gene. Neuromuscul. Disord..

[17] Yi H (2017). Antibody-mediated enzyme replacement therapy targeting both lysosomal and cytoplasmic glycogen in Pompe disease. J. Mol. Med..

[18] Lee NC (2020). Ultrastructural and diffusion tensor imaging studies reveal axon abnormalities in Pompe disease mice. Sci. Rep..

[19] Almodovar-Paya A (2020). Preclinical research in glycogen storage diseases: A comprehensive review of current animal models. Int. J. Mol. Sci..

[20] Sidman RL (2008). Temporal neuropathologic and behavioral phenotype of 6neo/6neo Pompe disease mice. J. Neuropathol. Exp. Neurol..

[21] Pitzer C, Kurpiers B, Eltokhi A (2021). Gait performance of adolescent mice assessed by the CatWalk XT depends on age, strain and sex and correlates with speed and body weight. Sci. Rep..

[22] Falk DJ (2015). Peripheral nerve and neuromuscular junction pathology in Pompe disease. Hum. Mol. Genet..

[23] Fukuda T (2006). Autophagy and lysosomes in Pompe disease. Autophagy.

[24] Yong Y, Hunter-Chang S, Stepanova E, Deppmann C (2021). Axonal spheroids in neurodegeneration. Mol. Cell. Neurosci..

[25] Watkins B, Schuster HM, Gerwin L, Schoser B, Kröger S (2022). The effect of methocarbamol and mexiletine on murine muscle spindle function. Muscle Nerve.

[26] Bornstein B (2023). Molecular characterization of the intact mouse muscle spindle using a multi-omics approach. eLife.

[27] Raben N (2003). Enzyme replacement therapy in the mouse model of Pompe disease. Mol. Genet. Metab..

[28] Fukuda T (2006). Dysfunction of endocytic and autophagic pathways in a lysosomal storage disease. Ann. Neurol..

[29] Kabeya Y (2000). LC3, a mammalian homologue of yeast Apg8p, is localized in autophagosome membranes after processing. EMBO J..

[30] Raben N (2001). Conditional tissue-specific expression of the acid alpha-glucosidase (GAA) gene in the GAA knockout mice: Implications for therapy. Hum. Mol. Genet..

[31] Byrne BJ (2011). Pompe disease: Design, methodology, and early findings from the Pompe registry. Mol. Genet. Metab..

[32] Oda K, Yamazaki K, Miura H, Shibasaki H, Kikuchi T (1992). Dying back type axonal degeneration of sensory nerve terminals in muscle spindles of the gracile axonal dystrophy (GAD) mutant mouse. Neuropathol. Appl. Neurobiol..

[33] Smeyne RJ (1994). Severe sensory and sympathetic neuropathies in mice carrying a disrupted Trk/NGF receptor gene. Nature.

[34] Ernfors P, Lee KF, Kucera J, Jaenisch R (1994). Lack of neurotrophin-3 leads to deficiencies in the peripheral nervous system and loss of limb proprioceptive afferents. Cell.

[35] Klein R (1994). Disruption of the neurotrophin-3 receptor gene trkC eliminates la muscle afferents and results in abnormal movements. Nature.

[36] Gambetti P, DiMauro S, Baker L (1971). Nervous system in Pompe's disease. Ultrastructure and biochemistry. J. Neuropathol. Exp. Neurol..

[37] Martin JJ, de Barsy T, van Hoof F, Palladini G (1973). Pompe's disease: an inborn lysosomal disorder with storage of glycogen. A study of brain and striated muscle. Acta Neuropathol..

[38] Lamartine SMM, Remiche G (2019). Late-onset Pompe disease associated with polyneuropathy. Neuromuscul. Disord..

[39] Tsai LK, Hwu WL, Lee NC, Huang PH, Chien YH (2019). Clinical features of Pompe disease with motor neuronopathy. Neuromuscul. Disord..

[40] Cheret C (2013). Bace1 and Neuregulin-1 cooperate to control formation and maintenance of muscle spindles. EMBO J..

[41] Ochoa J, Torebjork HE, Culp WJ, Schady W (1982). Abnormal spontaneous activity in single sensory nerve fibers in humans. Muscle Nerve.

[42] Nordin M, Nystrom B, Wallin U, Hagbarth KE (1984). Ectopic sensory discharges and paresthesiae in patients with disorders of peripheral nerves, dorsal roots and dorsal columns. Pain.

[43] Fagoe ND (2016). Evaluation of five tests for sensitivity to functional deficits following cervical or thoracic dorsal column transection in the rat. PLoS ONE.

[44] Mayer WP, Akay T (2021). The role of muscle spindle feedback in the guidance of hindlimb movement by the ipsilateral forelimb during locomotion in mice. eNeuro.

[45] van der Walt JD, Swash M, Leake J, Cox EL (1987). The pattern of involvement of adult-onset acid maltase deficiency at autopsy. Muscle Nerve.

[46] Horlings CG, van Engelen BG, Allum JH, Bloem BR (2008). A weak balance: The contribution of muscle weakness to postural instability and falls. Nat. Clin. Pract. Neurol..

[47] Horlings CG (2009). Balance control in patients with distal versus proximal muscle weakness. Neuroscience.

[48] Aman JE, Elangovan N, Yeh IL, Konczak J (2014). The effectiveness of proprioceptive training for improving motor function: A systematic review. Front. Hum. Neurosci..

[49] Yong MS, Lee YS (2017). Effect of ankle proprioceptive exercise on static and dynamic balance in normal adults. J. Phys. Ther. Sci..

[50] Iolascon G (2020). Adapted physical activity and therapeutic exercise in late-onset Pompe disease (LOPD): A two-step rehabilitative approach. Neurol. Sci..

[51] Montagnese F, Thiele S, Wenninger S, Schoser B (2016). Long-term whole-body vibration training in two late-onset Pompe disease patients. Neurol. Sci..

[52] Schaaf GJ (2015). Lack of robust satellite cell activation and muscle regeneration during the progression of Pompe disease. Acta Neuropathol. Commun..

[53] Hintze S (2021). Uptake of moss-derived human recombinant GAA in Gaa (−/−) mice. JIMD Rep..

[54] Garrick JM, Costa LG, Cole TB, Marsillach J (2021). Evaluating gait and locomotion in rodents with the CatWalk. Curr. Protoc..

[55] Mock JT (2018). Gait analyses in mice: Effects of age and glutathione deficiency. Aging Dis..

[56] Brooks SP, Dunnett SB (2009). Tests to assess motor phenotype in mice: A user's guide. Nature Rev. Neurosci..

[57] Batka RJ (2014). The need for speed in rodent locomotion analyses. Anat. Rec..

[58] Wilkinson KA, Kloefkorn HE, Hochman S (2012). Characterization of muscle spindle afferents in the adult mouse using an in vitro muscle-nerve preparation. PLoS ONE.

[59] Franco JA, Kloefkorn HE, Hochman S, Wilkinson KA (2014). An in vitro adult mouse muscle-nerve preparation for studying the firing properties of muscle afferents. J. Vis. Exp..

[60] Gerwin L, Haupt C, Wilkinson KA, Kröger S (2019). Acetylcholine receptors in the equatorial region of intrafusal muscle fibres modulate mouse muscle spindle sensitivity. J. Physiol..

[61] Gerwin L (2020). Impaired muscle spindle function in murine models of muscular dystrophy. J. Physiol..

[62] Than K (2021). Vesicle-released glutamate is necessary to maintain muscle spindle afferent excitability but not dynamic sensitivity in adult mice. J. Physiol..

[63] Larsson L, Edstrom L (1986). Effects of age on enzyme-histochemical fibre spectra and contractile properties of fast- and slow-twitch skeletal muscles in the rat. J. Neurol. Sci..

[64] Brooks SV, Faulkner JA (1988). Contractile properties of skeletal muscles from young, adult and aged mice. J. Physiol..

[65] Zhang Y, Wesolowski M, Karakatsani A, Witzemann V, Kröger S (2014). Formation of cholinergic synapse-like specializations at developing murine muscle spindles. Dev. Biol..

[66] Miller JB, Crow MT, Stockdale FE (1985). Slow and fast myosin heavy chain content defines three types of myotubes in early muscle cell cultures. J. Cell Biol..

[67] Pedrosa F, Soukup T, Thornell LE (1990). Expression of an alpha cardiac-like myosin heavy chain in muscle spindle fibres. Histochemistry.

[68] Kucera J, Walro JM, Gorza L (1992). Expression of type-specific MHC isoforms in rat intrafusal muscle fibers. J. Histochem. Cytochem..

[69] Walro JM, Kucera J (1999). Why adult mammalian intrafusal and extrafusal fibers contain different myosin heavy-chain isoforms. Trends Neurosci..

[70] Lionikas A (2013). Analyses of muscle spindles in the soleus of six inbred mouse strains. J. Anat..

[71] Sonner MJ, Walters MC, Ladle DR (2017). Analysis of proprioceptive sensory innervation of the mouse soleus: A whole-mount muscle approach. PLoS ONE.

[72] Schneider CA, Rasband WS, Eliceiri KW (2012). NIH Image to ImageJ: 25 years of image analysis. Nat. Method.

