# Author Correction: Degeneration of muscle spindles in a murine model of Pompe disease

**DOI:** 10.1038/s41598-024-63491-0

**Published:** 2024-06-11

**Authors:** Bridgette Watkins, Jürgen Schultheiß, Andi Rafuna, Stefan Hintze, Peter Meinke, Benedikt Schoser, Stephan Kröger

**Affiliations:** 1 Department of Physiological Genomics, Biomedical Center, Ludwig-Maximilians-University, Grosshaderner Strasse 9, 82152 Planegg-Martinsried, Germany; 2 Department of Neurology, Friedrich-Baur-Institute, LMU Klinikum, Ludwig-Maximilians-University, Munich, Germany

Correction to: *Scientific Reports* 10.1038/s41598-023-33543-y, published online 21 April 2023

The original version of this Article contained errors in Figure 1, where panel (G) was a duplication of panel (**D**) algorithms for Lateral Displacement and Print Position post initial data collection. The original Figure 1 and accompanying legend appear below.

**Figure 1:**
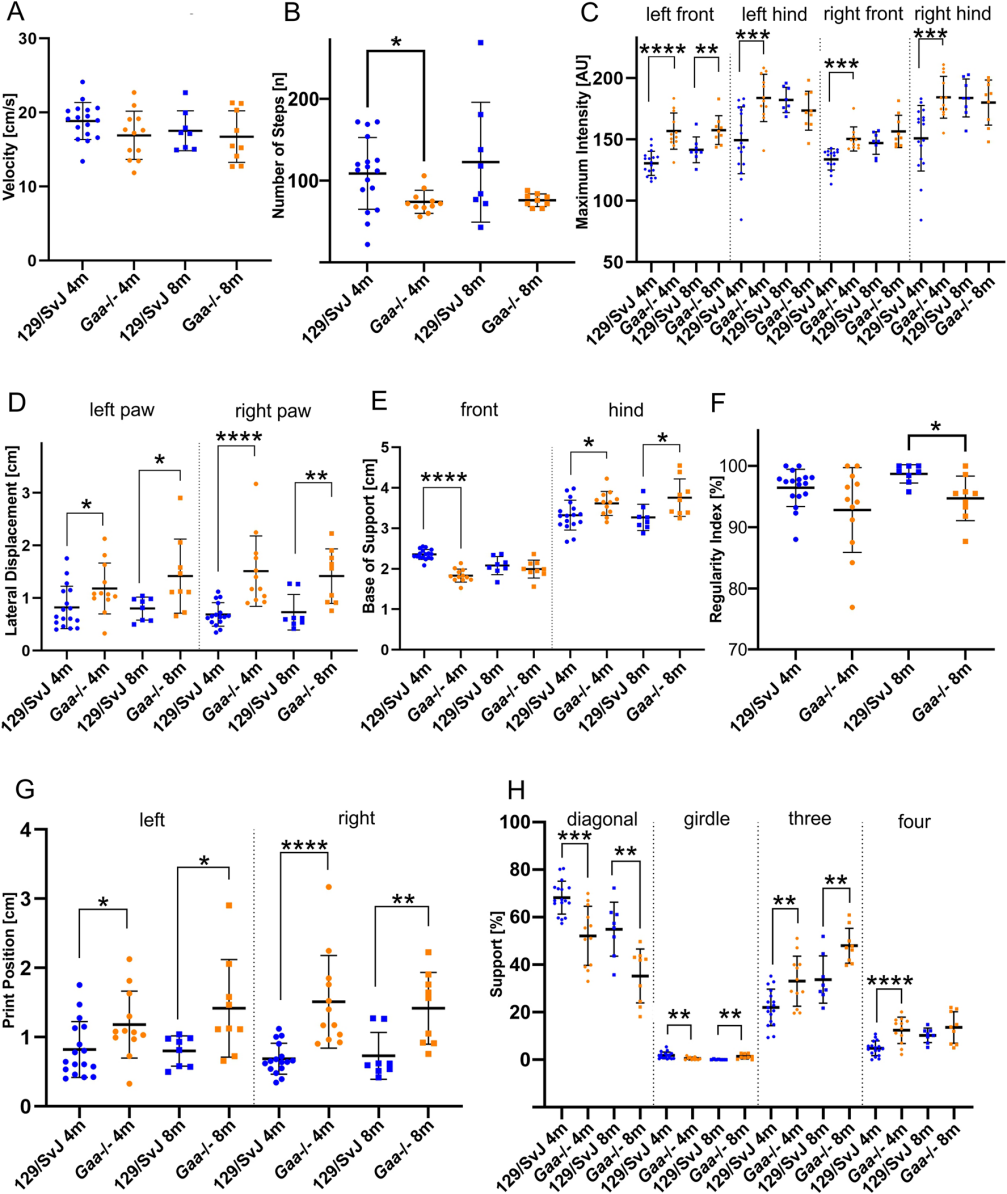
*Gaa*^−/−^ mice have an abnormal motor coordination. Automatic gait analysis revealed that many general locomotor parameters were similar in wildtype 129/SvJ mice (blue dots) and *Gaa*^−/−^ mice (orange dots), including velocity of movement (**A**) and number of steps ((**B**); the slightly reduced number of in 4-month-old mice is most likely due to the weight difference). Other parameters are different between *Gaa*^−/−^ mice and age-matched control mice due to their different muscle force, including the maximum intensity of the footprints (**C**). On the other hand, both mouse lines behaved differently with respect to locomotion coordination parameters, including the lateral displacement (**D**), the base of support for the front- and hind limbs (**E**), as well as the regularity index (**F**). The distance between the position of the hind paw and the position of the previously placed front paw on the same side of the body and in the same step cycle (print position) was increased on the left as well as on the right side in *Gaa*^−/−^ mice (**G**) at both ages examined. Moreover, the time the mice were supported by contacting the ground with the diagonal and girdle sides limbs as well as the time the animal was supported by three or four limbs was longer in *Gaa*^−/−^ mice compared to 129/SvJ control mice (**H**). For a complete list of gait parameters analyzed see Supplementary Table 1. The bars show the mean ± SD with N = 17 (4-month-old 129SvJ) and N = 12 (4-month-old *Gaa*^−/−^), N = 8 (8-month-old 129SvJ), N = 9 (8-month old *Gaa*^−/−^) mice. Statistical significance was calculated using the unpaired student’s t-test.

As a result of this error, Figure 1 legend,

“*Gaa*^−/−^ mice have an abnormal motor coordination. Automatic gait analysis revealed that many general locomotor parameters were similar in wildtype 129/SvJ mice (blue dots) and *Gaa*^−/−^ mice (orange dots), including velocity of movement (**A**) and number of steps ((**B**); the slightly reduced number of in 4-month-old mice is most likely due to the weight difference). Other parameters are different between *Gaa*^−/−^ mice and age-matched control mice due to their different muscle force, including the maximum intensity of the footprints (**C**). On the other hand, both mouse lines behaved differently with respect to locomotion coordination parameters, including the lateral displacement (**D**), the base of support for the front- and hind limbs (**E**), as well as the regularity index (**F**). The distance between the position of the hind paw and the position of the previously placed front paw on the same side of the body and in the same step cycle (print position) was increased on the left as well as on the right side in *Gaa*^−/−^ mice (**G**) at both ages examined. Moreover, the time the mice were supported by contacting the ground with the diagonal and girdle sides limbs as well as the time the animal was supported by three or four limbs was longer in *Gaa*^−/−^ mice compared to 129/SvJ control mice (**H**). For a complete list of gait parameters analyzed see Supplementary Table 1. The bars show the mean ± SD with N = 17 (4-month-old 129SvJ) and N = 12 (4-month-old *Gaa*^−/−^), N = 8 (8-month-old 129SvJ), N = 9 (8-month old *Gaa*^−/−^) mice. Statistical significance was calculated using the unpaired student’s t-test.”

now reads:

“*Gaa*^-/-^ mice have an abnormal motor coordination. Automatic gait analysis revealed that many general locomotor parameters were similar in wildtype 129/SvJ mice (blue dots) and *Gaa*^-/-^ mice (orange dots), including velocity of movement (A) and number of steps (B; the slightly reduced number of steps in 4-month-old mice is most likely due to the weight difference). Other parameters are different between *Gaa*^-/-^ mice and age-matched control mice due to their different muscle force, including the maximum intensity of the footprints (C). On the other hand, both mouse lines behaved differently with respect to locomotion coordination parameters, including the print position (the distance between the position of the hind paw and the position of the previously placed front paw on the same side of the body and in the same step cycle; D), the base of support for the front- and hind limbs (E), as well as the regularity index (F). Moreover, the time the mice were supported by contacting the ground with the diagonal and girdle sides limbs as well as the time the animal was supported by three or four limbs was longer in *Gaa*^-/-^ mice compared to 129/SvJ control mice (G). For a complete list of gait parameters analyzed see Supplementary Table 1. The bars show the mean +/- SD with N=17 (4-month-old 129SvJ) and N=12 (4-month-old *Gaa*^-/-^), N=8 (8-month-old 129SvJ), N=9 (8-month old *Gaa*^-/-^) mice. Statistical significance was calculated using the unpaired student’s t-test.”

Additionally, in the Results, under the subheading ‘***Gaa*** ^−/−^ mice have motor coordination deficits’,

“These parameters include the lateral displacement (distance between the position of the hind paw and the position of the previously placed front paw on the same side of the body (ipsilateral) and in the same step cycle; Fig. 1D) and the wider base of support particular of the hind limbs (distance between two hind paws, Fig. 1E).”

now reads:

“These parameters include the print position (distance between the position of the hind paw and the position of the previously placed front paw on the same side of the body (ipsilateral) and in the same step cycle; Fig. 1D), which was increased on both sides and at both ages in *Gaa*^-/-^ mice. In addition, the base of support (average width between two paws) particular of the hind limbs was increased (Fig. 1E).”

“The print position (distance between the position of the hind paw and the position of the previously placed front paw on the same side of the body (ipsilateral) and in the same step cycle) was increased on both sides of the body and at both ages in *Gaa*^−/−^ mice (Fig. 1G). The relative duration of the simultaneous contact with the glass plate of all combinations of paws is another parameter, which differed significantly between *Gaa*^−/−^ and control mice at both ages analyzed (Fig. 1H).”

now reads:

“The relative duration of the simultaneous contact with the glass plate of all combinations of paws is another parameter, which differed significantly between *Gaa*^-/-^ and control mice at both ages analyzed (Fig. 1G).”

“In contrast, the time each animal was supported by simultaneous contact of the diagonal pair of paws (right front paw and left hind paw or left front paw and right hind paw) were significantly lower in *Gaa*^−/−^ mice compared to wildtype mice (Fig. 1H). The time of support for the girdle paws (right front paw and left front paw or right hind paw and left hind paw) was lower in 4-month-old and higher in 8-month-old *Gaa*^−/−^ mice (Fig. 1H). Moreover, the relative amount of time the animal simultaneously spent on three or four paws was higher in *Gaa*^−/−^ mice compared to age-matched control mice (Fig. 1H).”

now reads:

“In contrast, the time each animal was supported by simultaneous contact of the diagonal pair of paws (right front paw and left hind paw or left front paw and right hind paw) were significantly lower in *Gaa*^-/-^ mice compared to wildtype mice (Fig. 1G). The time of support for the girdle paws (right front paw and left front paw or right hind paw and left hind paw) was lower in 4-month-old and higher in 8-month-old *Gaa*^-/-^ mice (Fig. 1G). Moreover, the relative amount of time the animal simultaneously spent on three or four paws was higher in *Gaa*^-/-^ mice compared to age-matched control mice (Fig. 1G).”

In the Discussion,

“The lateral displacement results, increased stance time and increase in hind base of support in *Gaa*^−/−^ mice are similar to the motor symptoms in patients.”

now reads:

“The print position results, increased stance time and increase in hind base of support in *Gaa*^-/-^ mice are similar to the motor symptoms in patients.”

The original Article has been corrected.

